# Joint longitudinal model-based meta-analysis of FEV_1_ and exacerbation rate in randomized COPD trials

**DOI:** 10.1007/s10928-023-09853-z

**Published:** 2023-03-22

**Authors:** Carolina Llanos-Paez, Claire Ambery, Shuying Yang, Misba Beerahee, Elodie L. Plan, Mats O. Karlsson

**Affiliations:** 1 Department of Pharmacy, Uppsala University, Uppsala, Sweden; 2 Clinical Pharmacology Modelling and Simulation, GSK, London, UK; 3 Department of Pharmacy, Uppsala University, BMC, Box 580, 751 23 Uppsala, Sweden

**Keywords:** Chronic obstructive pulmonary disease, Model-based meta-analysis, Forced expiratory volume in one second, Exacerbation rate

## Abstract

Model-based meta-analysis (MBMA) is an approach that integrates relevant summary level data from heterogeneously designed randomized controlled trials (RCTs). This study not only evaluated the predictability of a published MBMA for forced expiratory volume in one second (FEV_1_) and its link to annual exacerbation rate in patients with chronic obstructive pulmonary disease (COPD) but also included data from new RCTs. A comparative effectiveness analysis across all drugs was also performed. Aggregated level data were collected from RCTs published between July 2013 and November 2020 (n = 132 references comprising 156 studies) and combined with data used in the legacy MBMA (published RCTs up to July 2013 - n = 142). The augmented data (n = 298) were used to evaluate the predictive performance of the published MBMA using goodness-of-fit plots for assessment. Furthermore, the model was extended including drugs that were not available before July 2013, estimating a new set of parameters. The legacy MBMA model predicted the post-2013 FEV_1_ data well, and new estimated parameters were similar to those of drugs in the same class. However, the exacerbation model overpredicted the post-2013 mean annual exacerbation rate data. Inclusion of year when the study started on the pre-treatment placebo rate improved the model predictive performance perhaps explaining potential improvements in the disease management over time. The addition of new data to the legacy COPD MBMA enabled a more robust model with increased predictability performance for both endpoints FEV_1_ and mean annual exacerbation rate.

**Supplementary Information:**

The online version contains supplementary material available at 10.1007/s10928-023-09853-z.

## Introduction

Model-based meta-analysis (MBMA) is a valuable tool that provides a quantitative framework for decision-making during drug development. Specifically, a MBMA typically integrates relevant summary level patient data (e.g., efficacy and/or safety data) from treatment arms of identified randomized controlled trials (RCTs), applying pharmacological models (e.g., dose/exposure response), for overall assessment of efficacy and safety [1]. During drug development, comparative effectiveness is important in decision-making; however, conducting head-to-head trials to benchmark against competitors is impractical, expensive, and time-consuming. One of the advantages of using a MBMA, compared to conventional meta-analysis, resides in the use of less restrictive inclusion/exclusion criteria for study selection, since it can characterize the response of interest as a parametric function of time integrating information from RCTs with different designs. This means that MBMA allows the direct comparison of treatment effects in silico, even in the absence of real-life head-to-head trials, taking into account RCTs heterogeneity (e.g., patient population characteristics or trial features) through quantification of inter-study variability (ISV) and inter-arm variability (IAV) [2]. Several longitudinal MBMA models have been developed across different therapeutic areas, including rheumatoid arthritis [3, 4], psoriasis [5], osteoporosis [6] and chronic obstructive pulmonary disease (COPD) [7], amongst others [2].

COPD is an inflammatory lung disease characterized by not fully reversible airflow obstruction. According to the World Health Organization estimate, COPD is the third leading cause of death worldwide, causing 3.23 million deaths in 2019 [8]. The Global Initiative for Chronic Obstructive Lung Disease (GOLD) [9] strategy recommends the use of short or long-acting β2 agonists (SABA and LABA, respectively), short or long-acting anticholinergic (SAMA and LAAC, respectively) or combined therapy with LABA/LAAC with/without inhaled corticosteroids (ICS). Some of the most used endpoints to assess disease progression, and hence drug effect, in COPD clinical trials include forced expiratory volume in one second (FEV_1_) and exacerbation rate, amongst others (alongside patient reported outcomes).

A legacy MBMA in COPD patients, which relates patient and trial characteristics and dosing to FEV_1_, and exacerbation rate summary level data, has been developed [7, 10]. This analysis included RCTs published up until 2013 with bronchodilators and anti-inflammatories given either as mono- (82%), dual-(17%), or triple-therapy (1%) combinations. However, more recent studies [11, 12] have focused on comparing the efficacy of the inhaled triple-therapy (with the addition of ICS). For example, a phase 3 trial [11] with more than 10,000 patients prospectively identified that once daily triple-therapy [umeclidinium (UMEC)/vilanterol (VI)/fluticasone furoate (FF)] was associated with a greater reduction in exacerbation rate than either of the dual-therapies UMEC/VI or VI/FF. Thus, the legacy MBMA model can be updated to include all the new trial data published since 2013; and be used to assess comparative effectiveness of new and established COPD maintenance treatments.

This analysis aims to (i) evaluate the application and predictability of a published longitudinal MBMA COPD model for FEV_1_ [7] and exacerbation rate [10], (ii) update the legacy MBMA with new information (e.g., new drugs and additional dual and triple combination studies), and therefore to address more contemporary questions using a more well-informed model, and (iii) perform a comparative effectiveness analysis across all drugs included in the analysis.

## Methods

### Data

The literature search, study selection, data extraction, processing and analysis were performed following the described Methods in the legacy MBMA [7]. The augmented data defined as the combination of both the new additional collected data (post-2013) and the legacy data (pre-2013) were used to perform the analysis.

#### *Literature search and study selection*

An automated literature search was conducted in November 2020 using OVID MEDLINE and Embase databases with a restriction on English language publications. Searching criteria comprised studies published between July 1, 2013 and November 24, 2020 using keywords such as “FEV_1_”, “COPD”, and related terms as well as the generic and brand names of established or under development long-acting bronchodilators and anti-inflammatory compounds used in the treatment of COPD. After removing duplicates, abstracts of potentially relevant articles were screened. Subsequently, full-text versions of the relevant identified articles were reviewed independently by C.L-P, SY, CA and MB.

Key selection criteria for the reviewed articles included (i) single- or double- blind multiple-dosing COPD maintenance trials (with the exception of the open-label triotropium Spiriva arms); (ii) a minimum of 30 and 50 patients for cross-over and parallel-group designs, respectively; and (iii) reported absolute morning trough FEV_1_ values. Change from baseline (CFB), placebo or comparator arm FEV_1_ observations were included only if they could be back-calculated to absolute values. For studies where the CFB in trough FEV_1_ could be transformed to absolute value (absolute FEV_1_ = FEV_1_ baseline + CFB) but the FEV_1_ baseline value was measured post-short acting bronchodilator (post-SABD), a correction was made (PostBD_correction_) on the transformed absolute morning trough FEV_1_ by estimating a correction factor (θ_correction_) (Eq. 1). Pre-dose FEV_1_ observations with respect to study drug but measured post-SABD were also included in the data for analysis. For the predictions of exacerbation rate, the mean annual rate of moderate or severe exacerbations per patient per year was used as the outcome of interest for analysis.

1 $${\text{P}\text{o}\text{s}\text{t}\text{B}\text{D}}_{\text{c}\text{o}\text{r}\text{r}\text{e}\text{c}\text{t}\text{i}\text{o}\text{n}}=\text{a}\text{b}\text{s}\text{o}\text{l}\text{u}\text{t}\text{e} {\text{F}\text{E}\text{V}}_{1\text{i},\text{j}}\cdot \left(1-{{\uptheta }}_{\text{c}\text{o}\text{r}\text{r}\text{e}\text{c}\text{t}\text{i}\text{o}\text{n}}\right)$$

#### Data extraction, processing, and analysis

Study characteristics data such as study size, inclusion criteria (e.g., exacerbation history and disease severity), treatment information and population demographic characteristics were extracted from the selected papers and collected using Microsoft Excel software. Relevant information that was not provided in the article was obtained from ClinicalTrials.gov (https://clinicaltrials.gov/), or databases from the different companies such as GSK (https://www.gsk-studyregister.com/en/), Boehringer Ingelheim (https://www.mystudywindow.com/), Novartis (https://www.novctrd.com/#/) and AstraZeneca (https://astrazenecagrouptrials.pharmacm.com/ST/Submission/Search). Data management and graphical exploration were performed in R software (The R Foundation for Statistical Computing) version 3.5.2 [13] using R packages [i.e. Xpose4 (http://xpose.sourceforge.net, version 4.6.1)] [14, 15]. Data analysis and modelling were performed in NONMEM software (ICON Development Solutions, Ellicott City, Maryland) version 7.5 together with an Intel FORTRAN compiler and Perl-speaks-NONMEM (PsN) version 5.2.6 [16].

Missing covariate values such mean age and any unknown fraction of patients who had a medication history and/or received background treatment during the run-in and/or study period were imputed as described in the legacy MBMA [7]. Specifically, multiple regression linear models reported in [7] were considered in the analysis.

### Model-based longitudinal meta-analysis

The same structural model components and variability from the legacy models were considered in this analysis to assess the predictive performance of the published MBMA [7] and exacerbation rate model [10]. Key structural components of the legacy MBMA model [7] used in this analysis are described below.

#### Structural meta-model for FEV_1_

The observed absolute morning trough FEV_1_ (L) for the *j*^*th*^ arm of the *i*^*th*^ study over time is described in Eq. 2. Sub-models were used to describe the model-predicted untreated study arm baseline FEV_1_ (B) (i.e., the pre-dose baseline at randomization), long-term disease progression (DP), placebo effect (PBO), and drug effects (E) of the background and study drug treatments (X). The residual unexplained variability (RUV) was assumed to follow a *N*(0, σ^2^) and weighted by the inverse of the square-root of the number of patients in the study arm (N).

2 $${\text{F}\text{E}\text{V}}_{1\text{i},\text{j}}\left(\text{t}\right)={\text{B}}_{\text{i},\text{j}}\left(\text{C}\text{O}\text{V}\right)+{\text{D}\text{P}}_{\text{i},\text{j}}\left(\text{t},{\text{B}}_{\text{i},\text{j}}\right)+{\text{P}\text{B}\text{O}}_{\text{i}}\left(\text{t}\right)+{\text{E}}_{\text{i},\text{j}}\left(\text{t},\text{X},{\text{B}}_{\text{i},\text{j}}\right)+{\text{P}\text{o}\text{s}\text{t}\text{B}\text{D}}_{\text{c}\text{o}\text{r}\text{r}\text{e}\text{c}\text{t}\text{i}\text{o}\text{n}}+\frac{{\upepsilon }}{\sqrt{{\text{N}}_{\text{i},\text{j}}}}$$

Covariate (COV) effects such as disease severity, exacerbation history (according to study protocol) and mean age at enrollment were included on B (Eq. 3).

3 $${\text{B}}_{\text{i},\text{j}}=\text{T}\text{V}\text{B}\cdot \left(1+{{\uptheta }}_{\text{C}\text{O}\text{V}}\cdot \left({\text{C}\text{O}\text{V}}_{\text{i},\text{j}}-{\text{C}\text{O}\text{V}}_{\text{m}\text{e}\text{d}}\right)\right)\cdot \text{e}\text{x}\text{p}\left({{\upeta }}_{\text{B}\text{i}}+\frac{{{\upkappa }}_{\text{i},\text{j}}}{\sqrt{\frac{{\text{N}}_{\text{i},\text{j}}}{200}}}\right)$$

Here, TVB is the estimated typical baseline value, θ_COV_ is the estimated covariate coefficient of the COV, COV_i,j_ is the observed mean covariate value in the study arm, and COV_med_ is the reported median of the mean covariate values across all study arms reported in the legacy MBMA, specifically a value of 63.6 years for age; two/four for the lowest/highest disease severity class; and one/zero for those studies that require/do not require patient having an exacerbation history to be enrolled. Random effects, η_B_ and κ, on baseline were included to describe inter-study variability (ISV) and inter-arm variability (IAV), respectively. IAV was weighted by the inverse of the square-root of N normalized to 200 patients, as used previously [7], which is close to the typical number of patients per study arm (Eq. 3).

Disease progression (DP) (Eq. 4) and placebo (PBO) (Eq. 5) models were described by linear and mixture models, respectively, as described in the legacy MBMA [7]. TVDP_slope_ is the estimated typical value for the DP slope for a typical 1.2 L baseline, and η_DPi_ is the random effect for ISV on DP. The mixture model was used to describe two subgroups of studies: those with a gradual PBO effect onset following an E_max_ model or studies with an immediate PBO effect onset. The estimated PT_50_ parameter indicates the time when the half-maximum PBO effect is reached in studies with a gradual onset, whereas it was fixed to 0.0001 weeks in studies with an immediate onset. The proportion of studies belonging to either subgroup was determined by using the $MIXTURE functionality in NONMEM. TVPBO_max_ is the estimated typical value of the maximum PBO effect, and η_PBO_ is the random effect for ISV on the maximum PBO effect.

4 $${\text{D}\text{P}}_{\text{i},\text{j}}={\text{T}\text{V}\text{D}\text{P}}_{\text{s}\text{l}\text{o}\text{p}\text{e}}\cdot \frac{{\text{B}}_{\text{i},\text{j}}}{1.2}\cdot \text{t}\cdot \text{e}\text{x}\text{p}\left({{\upeta }}_{\text{D}\text{P}\text{i}}\right)$$

5 $${\text{P}\text{B}\text{O}}_{\text{i}}\left(\text{t}\right)=\left({\text{T}\text{V}\text{P}\text{B}\text{O}}_{\text{m}\text{a}\text{x}}+{{\upeta }}_{\text{P}\text{B}\text{O}\text{i}}\right)\cdot \frac{\text{t}}{{\text{P}\text{T}}_{50}+\text{t}}$$

For each compound available in the dataset, an individual drug effect (E_x_) was determined. If data were available, dose-response information for a compound was described using an E_max_ model (Eq. 6), where D_xi,j_ is the dose of compound x in the *j*^*th*^ study arm of the *i*^*th*^ study, and ED_50,x_ is the estimated dose resulting in half-maximum efficacy. The maximum efficacy (E_max_) was calculated as shown in Eq. 7 based on the estimated efficacy (Eff_ref,x_) of the reference dose (D_ref,x_) and ED_50,x_. For those compounds where ED_50,x_ was unidentifiable, it was assumed that the compound has the same efficacy at all dose levels present in the dataset. For some compounds of the same class, some parameters were constrained to be the same. Specifically, aformoterol and formoterol share the same E_max_ as well as tiotropium (blinded) and tiotropium Respimat. The ED_50_ parameter for aformoterol was constrained to be half the ED_50_ value of formoterol, whereas the ED_50_ for tiotropium (blinded) was constrained to be equal to the ED_50_ of tiotropium (open-label).

6 $${\text{E}}_{\text{x}\text{i},\text{j}}=\frac{{\text{E}}_{\text{m}\text{a}\text{x},\text{x}}\cdot {\text{D}}_{\text{x}\text{i},\text{j}}}{{\text{D}}_{\text{x}\text{i},\text{j}}+{\text{E}\text{D}}_{50,\text{x}}}$$

7 $${\text{E}}_{\text{m}\text{a}\text{x},\text{x}}={\text{E}\text{f}\text{f}}_{\text{r}\text{e}\text{f},\text{x}}\cdot \frac{\left({\text{D}}_{\text{r}\text{e}\text{f},\text{x}}+{\text{E}\text{D}}_{50,\text{x}}\right)}{{\text{D}}_{\text{r}\text{e}\text{f},\text{x}}}$$

Treatment effects for the COPD medication received as background treatment during run-in and post-randomization, as well as a treatment class-dependent time course of effect onset were included in the model [7]. In addition, the effect of B_i,j_ (the model-predicted untreated study arm baseline) and ISV on the overall effect of bronchodilators (E_BD_) and anti-inflammatory (E_AI_) treatments were considered using a sub-model described in Eq. 8, where η_BD/AI i_ is the random effect for ISV on the bronchodilators/anti-inflammatory treatments, and BE_BD/AI i,j_ is the interacting effect of the B_i,j_ described in Eq. 9. A step function (STEP_B_) was used to describe this effect with B_i,j_ below 1.2 L (more severe scenario) resulting in an estimated reduction in the effect of drug treatment (θ_B_^BD/AI^) [7].

8 $${\text{E}}_{\text{B}\text{D}/\text{A}\text{I}\text{i},\text{j}\left(\text{t}\right)}={\text{E}}_{\text{B}\text{D}/\text{A}\text{I}\text{i},\text{j}}\left(\text{t}\right)\cdot \left(1+{{\upeta }}_{\text{B}\text{D}/\text{A}\text{I}\text{i}}\right)\cdot {\text{B}\text{E}}_{\text{B}\text{D}/\text{A}\text{I}\text{i},\text{j}}$$

9 $${\text{B}\text{E}}_{\text{B}\text{D}/\text{A}\text{I}\text{i},\text{j}}=\left(1-{\text{S}\text{T}\text{E}\text{P}}_{\text{B}}\right)\cdot \left(1+{{\uptheta }}_{\text{B}}^{\text{B}\text{D}/\text{A}\text{I}}\cdot \left({\text{B}}_{\text{i},\text{j}}-1.2\right)\right)+{\text{S}\text{T}\text{E}\text{P}}_{\text{B}}$$

$${STEP}_{B}=\left\{\begin{array}{c}1 \,\,\,If\,{B}_{i,j}\ge 1.2 L\\ 0 \,\,\,If\,{B}_{i,j} <1.2 L\end{array}\right.$$

An empirical interaction model allowing an infra-additive interaction (i.e., combined effect of both drugs is less than the sum of the effects of either drug alone) determined by the long-acting bronchodilator interaction parameter in the legacy MBMA [7] was used to describe the interaction between bronchodilators of different drug classes (e.g., combinations of LABA and LAAC compounds), whereas for all other maintenance treatment combinations were assumed to have a fully additive effect. This means that additive or synergistic interactions for other plausible combinations (e.g., ICS/LABA) were not contemplated in the model. The interaction between SABD and long-acting bronchodilators for those FEV_1_ observations measured post-SABD was considered by estimating a fractional reduction in the overall bronchodilator effect [7].

#### MBMA prediction of exacerbation rate

A link between MBMA-predicted placebo adjusted bronchodilator and anti-inflammatory drug effect on FEV_1_ (ΔΔFEV_1BD_ and ΔΔFEV_1AI_, respectively) and annual rate of moderate-severe exacerbations at week 12 for arm *j*^th^ in study *i*^th^ (ER_predi,j_) has been previously described [10, 17]. The ER_predi,j_ (Eq. 10) is modeled as the product between pre-treatment exacerbation rate (ER_PBOi,j_) (Eq. 11 and Eq. 12) and the typical exacerbation rate ratio between treatment and placebo groups (TVR_i,j_) (Eq. 13).

10 $${\text{E}\text{R}}_{\text{p}\text{r}\text{e}{\text{d}}_{\text{i},\text{j}}}={\text{E}\text{R}}_{\text{P}\text{B}{\text{O}}_{\text{i},\text{j}}}\cdot {\text{T}\text{V}\text{R}}_{\text{i},\text{j}}$$

11 $${\text{T}\text{V}\text{E}\text{R}}_{\text{P}\text{B}{\text{O}}_{\text{i},\text{j}}}= \left\{\begin{array}{c}{{\uptheta }}_{\text{E}\text{R}\text{P}\text{B}\text{O}\text{i},\text{j}}\cdot \text{exp}\left(\left(\text{p}\text{p}{\text{F}\text{E}\text{V}1}_{{1}_{\text{i},\text{j}}}-41\right)\cdot {{\uptheta }}_{\text{E}\text{R}}+\left({\text{I}\text{C}\text{S}}_{{\text{p}}_{\text{i},\text{j}}}-61\right)\cdot {{\uptheta }}_{\text{H}\text{I}\text{C}\text{S}}\right) If\,{\text{E}}_{\text{H}\text{I}\text{S}\text{T}}\,is\,required\\ {{\uptheta }}_{\text{E}\text{R}\text{P}\text{B}\text{O}\text{i},\text{j}}\cdot \left(1+{{\uptheta }}_{{\text{E}}_{\text{H}\text{I}\text{S}\text{T}}}\right)\cdot \text{exp}\left(\left(\text{p}\text{p}{\text{F}\text{E}\text{V}1}_{{1}_{\text{i},\text{j}}}-49\right)\cdot {{\uptheta }}_{\text{E}\text{R}}+\left({\text{I}\text{C}\text{S}}_{{\text{p}}_{\text{i},\text{j}}}-51\right)\cdot {{\uptheta }}_{\text{H}\text{I}\text{C}\text{S}}\right) If\,{\text{E}}_{\text{H}\text{I}\text{S}\text{T}}\,is\,not\,required\end{array}\right.$$

12 $${\text{E}\text{R}}_{\text{P}\text{B}{\text{O}}_{\text{i},\text{j}}}= {\text{T}\text{V}\text{E}\text{R}}_{\text{P}\text{B}{\text{O}}_{\text{i},\text{j}}}\cdot \text{e}\text{x}\text{p}\left({{\upeta }}_{\text{E}\text{R}}\right)$$

13 $${\text{T}\text{V}\text{R}}_{\text{i},\text{j}}=\text{e}\text{x}\text{p}({{\uptheta }}_{\text{B}\text{D}}\cdot {\Delta }{\Delta }{\text{F}\text{E}\text{V}}_{{1}_{\text{B}\text{D}}}+ {\text{I}\text{C}\text{S}}_{\text{n}\text{a}\text{i}\text{v}\text{e}{\text{f}}_{\text{i},\text{j}}}\cdot {{\uptheta }}_{\text{B}\text{D}}\cdot {\Delta }{\Delta }{\text{F}\text{E}\text{V}}_{{1}_{\text{A}\text{I}}})\cdot (1-{\text{I}\text{C}\text{S}}_{\text{e}\text{x}\text{p}{\text{f}}_{\text{i},\text{j}}}\cdot {\varDelta \varDelta {\text{F}\text{E}\text{V}_1}_{\text{A}\text{I}}}^{{{\uptheta }}_{\text{A}\text{I}\text{e}\text{x}\text{p}}})$$

The TVER_PBOi,j_ is the estimated typical ER_PBOi,j_ value (θ_ERPBOi,j_) for a study that required or not a history of exacerbation (E_HIST_) and a washout of ICS, θ_ER_ is the estimated fractional change in ER_PBOi,j_ for 1% point increase in %predicted FEV_1_ (ppFEV_1i,j_), θ_EHIST_ is the estimated fractional change in ER_PBOi,j_ if history of exacerbations is not required and θ_HICS_ is the estimated fractional change in ER_PBOi,j_ for 1% point increase in medical history of ICS, with ICS_pi,j_ representing the percent of patients with a history of ICS usage, η_ER_ is the random effect for ISV on the ER_PBOi,j_.

The fraction of ICS-naïve patients (ICS_naivefi,j_) and fraction of ICS-experienced patients (ICS_expfi,j_) were predictors for TVR_i,j_ (Eq. 13), whereas θ_BD_ represents the estimated fractional change in TVR_i,j_ for each unit (L) of drug effect from a direct bronchodilator, and θ_AIexp_ is the estimated power function for TVR_i,j_ based on unit (L) of drug effect from an anti-inflammatory drug in patients with medical history of ICS.

Stochastic error (ɛ) due to limited sample size was incorporated in the model (Eq. 14), where ɛ follows a *N*(0, σ^2^) and N_i,j_ was the number of subjects for arm *j*^*th*^ in study *i*^*th*^ normalized to 500 patients which is close to the typical number of patients per treatment arm. The treatment duration in weeks (TRT_dur_) was also considered in the RUV using an estimated power function (θ_TRT_).

14 $$\text{l}\text{o}\text{g}\left({\text{E}\text{R}}_{\text{o}\text{b}{\text{s}}_{\text{i},\text{j}}}\right)=\text{l}\text{o}\text{g}\left({\text{E}\text{R}}_{\text{p}\text{r}\text{e}{\text{d}}_{\text{i},\text{j}}}\right)+\frac{1}{\sqrt{\frac{{\text{N}}_{\text{i},\text{j}}}{500}}}\cdot {\left(\left.\frac{{\text{T}\text{R}\text{T}}_{\text{d}\text{u}\text{r}}}{52}\right)\right.}^{{{\uptheta }}_{\text{T}\text{R}\text{T}}}\cdot {{\upepsilon }}_{\text{i},\text{j}}$$

#### Models’ predictive performance

The predictive performance of both published models for FEV_1_ [7] and exacerbation rate [10] using the augmented dataset was assessed visually using goodness-of-fit plots such as observed FEV_1_ vs. population/individual predictions, conditional weighted residuals (CWRES) vs. time and individual weighted residuals (iWRES) vs. individual predictions. To produce these plots, the model parameters from the published models were considered with number of objective function evaluations set to zero (MAXEVAL = 0). At this stage, changes to the models were considered if they showed a poor predictability based on trends in the goodness-of-fit plots that indicates whether a model is correct or not [18]. In case of including covariates, decision was based on parameter plausibility and uncertainty, goodness-of-fit plots, and objective function value (OFV), where the difference in OFV between two nested model approximates the chi-square (χ^2^) statistics, which can be tested for significance (χ^2^_1,0.05_ = 3.84).

#### Extension to new drugs

The published MBMA was extended with clinical trial data that were not available before July 2013. This adds information on new drugs and drug combinations, not present in the previous data base. The same treatment effect models described in Eq. 6 and Eq. 7 were applied for the new drugs, and a new set of parameter estimates was obtained. For ICS, LABA and LAAC compounds, a time-course of effect onset was tested as described previously [7] and retained in the model if a reduction in OFV was observed (regardless of statistical significance). In the published model, data from fluticasone propionate and FF were handled as a single drug “fluticasone” given either twice a day (b.i.d.) or once a day (q.d.), respectively. The efficacy parameter for fluticasone q.d. was estimated as a relative reference efficacy of fluticasone q.d. compared to b.i.d. In this analysis they were considered as different drugs and different set of parameters were estimated for each drug.

#### Comparative effectiveness

The comparative effectiveness for FEV_1_ across bronchodilators and anti-inflammatory compounds was assessed as described previously [17]. The drug effect parameters were re-parameterized as relative effects (R_effect_) of two drugs for all comparison of interest (Eq. 15).

15 $${\text{R}}_{\text{e}\text{f}\text{f}\text{e}\text{c}\text{t}}=\frac{{\text{E}}_{\text{d}\text{r}\text{u}\text{g}1}}{{\text{E}}_{\text{d}\text{r}\text{u}\text{g}2}}$$

Confidence intervals (CI) around the R_effect_ were obtained using the log-likelihood profiling tool (llp) in PsN. If R_effect_ is larger than one would mean that drug 1 is superior to drug 2, conversely if R_effect_ is smaller than one would mean that drug 1 is inferior to drug 2. If the CI for R_effect_ includes one, superiority or inferiority cannot be established.

## Results

### Data

The post-2013 dataset includes a total of 132 references comprising 156 studies (Table S1 in Supplementary material). Combined with the pre-2013 data, there is a total of 298 studies including 250,543 patients who contributed to 4,137 mean morning trough FEV_1_ observations for analysis. The augmented data include a mean of 274 patients per study arm (number of arms: 914). Fifty-two (17%) studies with a total of 99,296 patients reported annual exacerbation rate for each study arm contributing to a total of 135 observations for analysis. Studies characteristics and patient demographics for the pre-2013, post-2013 and augmented datasets are shown in Table 1.

A total of 23 compounds were given across 914 study arms as mono- (71%), dual- (25%) or triple-therapy (4%). Compounds and their dosing regimens are shown in Table 2.

**Table 1:** Characteristics of studies including in the analysis

Characteristic	Data 1996–2013 (n = 142)		Data 2013–2020 (n = 156)		Total (n = 298)	
	Mean	Median (range) [%missing]	Mean	Median (range) [% missing]	Mean	Median (range) [% missing]
Study duration (weeks)	28	16 (1.0–208) [0]	20.8	12 (1.0–120) [0]	24.2	12.0 (1–208) [0]
Total number of patients enrolled	749.5	557.5 (30–6112) [0]	1246	768.5 (43–20313)^a^ [0]	1010	672.5 (30–20313) [0]
Number of patients per study arm at baseline	264	185 (18–3006) [0]	282.7	196 (20–5724)^a^ [0]	274.1	193.5 (18–5724) [0]
Number of patients per study arm contributing to FEV_1_ observations	264	185 (18–3006) [0]	249.7	196 (20–3366)^b^ [0]	256.3	194 (18–3366) [0]
Number of patients per study arm contributing to ER observations	543.4	408.5 (198–3006) [0]	1015	526 (154–5724) [0]	735.5	412 (154–5724) [0]
Number of arms	2.95	2 (1–10) [0]	3.17	3 (2–8) [0]	3.01	3 (1–10) [0]
Males per study arm (%)	71.2	72 (43–100) [0]	64.8	64.0 (34.8–100) [0]	67.7	67.1 (34.8–100) [0]
Mean age per study arm (years)	63.4	63.6 (52.4–70.2) [1.0]	63.2	63.3 (54.6–71.5) [0]	63.3	63.5 (52.4–71.5) [0.4]
Smokers per study arm (%)	45.0	44.5 (0–100) [17.9]	48.5	49.1 (0–80) [8.6]	47.0	46.1 (0–100) [12.9]
Mean pack-years per study arm	46.5	46.0 (10.0–65.5) [23.4]	46.0	46.3 (18.7–72.3) [30.9]	46.2	46.1 (10–72.3) [27.4]
Mean relative reversibility (%)	13.6	13.9 (2.47–24.6) [29.1]	15.2	15.1 (4.10–27.7) [57.0]	14.3	14.4 (2.47–27.7) [44.3]
Mean %predicted FEV_1_ per study arm	48.9	48.6 (36.0–86.9) [0]	51.7	52.3 (32.9–78.1) [6.4]	50.4	51.3 (32.9–86.9) [3.49]
Patients with medication history per study arm						
ICS (%)	46.1	46.9 (0–100) [38.9]	44.4	45.3 (0–100) [23.8]	45.6	45.0 (0–100) [18.4]
LABA (%)	43.0	41.1 (0–100) [63.7]	44.6	44.4 (0–100) [43.2]	43.5	39.9 (0–100) [39.4]
LAAC (%)	19.1	17.2 (0–100) [74]	30.1	28.6 (0–100) [47.7]	22.9	19.0 (0–100) [49.4]
Patients with background medication during run-in per study arm						
ICS	32.0	34.2 (0–100) [28.2]	42.7	44.0 (0–100) [4.44]	39.7	43.7 (0–100) [5.3]
LABA	9.6	0 (0–100) [23.6]	19.4	0 (0–100) [17.9]	15.8	0 (0–100) [19.0]
LAAC	3.2	0 (0–100) [37.7]	8.6	0 (0–100) [23.0]	6.37	0 (0–100) [29.6]
Patients with background medication during the study period per study arm						
ICS	25.3	0 (0–100) [27.0]	38.8	43.9 (0–100) [6.5]	35.2	43.3 (0–100) [5.79]
LABA	5.0	0 (0–100) [21.0]	10.2	0 (0–100) [16.2]	8.56	0 (0–100) [17.4]
LAAC	0.8	0 (0–100) [36.5]	5.7	0 (0–100) [20.6]	3.70	0 (0–100) [27.8]

^a^Study NCT01126437 enrolled 20313 patients (https://www.mystudywindow.com/trial/completed/192691/0205-0452), with 17116 patients contributing FEV_1_ values at baseline, however, a subgroup of 1370 patients was considered for the analysis of through FEV_1_ during the study period

^b^number of patients contributing to FEV_1_ observations after baseline; *ER* exacerbation rate, *FEV*_1_ forced expiratory volume in one second; *ICS* inhaled corticosteroids; *LABA* long-acting bronchodilator; *LAAC* long-acting anticholinergics

**Table 2:** Compounds included in the analysis with their drug class and dosing regimen

Drug class	Compound	Dosing regimen (ug)
Long-acting beta-agonists (LABA)	Arformoterol b.i.d.	15, 25, 50
	Formoterol b.i.d.	4.5, 6, 7.2, 9, 9.6, 10, 12, 18, 20, 24
	Indacaterol q.d.	15, 50, 75, 85, 100, 110, 150, 200, 300, 400, 600, 800
	Salmeterol b.i.d.	42, 50, 100
	Vilanterol q.d.	3, 6.25, 12.5, 25, 50
	Olodaterol q.d.	2, 5, 10, 20
Long-acting anticholinergics (LAAC)	Aclidinium q.d.	25, 50, 100, 200, 400
	Aclidinium b.i.d.	100, 200, 400
	BEA2180 q.d.	50, 100, 200
	Glycopyrronium q.d.	12.5, 25, 43, 50, 100, 200
	Glycopyrronium b.i.d.	0.6, 1.2, 2.4, 3, 3.6, 4.6, 6.25, 7.2, 9, 10, 12.5, 14.4, 15.6, 18, 25, 28.8, 31.2, 36, 50, 72, 100
	GSK233705 b.i.d.	20, 50
	Tiotropium – Handihaler q.d.	4.5, 9, 18, 36
	Tiotropium – Respimat q.d.	1.25, 2.5, 5, 10, 20
	Umeclidinium q.d.	15.6, 31.25, 62.5, 125, 250, 500, 1000
	Umeclidinium b.i.d.	15.6, 31.25, 62.5, 125, 250
	Revefenacin q.d.	44, 88, 175, 350
Inhaled corticosteroids	Beclomethasone b.i.d	100, 200
	Budesonide b.i.d.	80, 160, 200, 320, 400, 800
	Fluticasone propionate b.i.d.	100, 230, 250, 500
	Fluticasone furoate q.d.	50, 100, 200, 400
	Mometasone q.d.	160, 800
	Mometasone b.i.d.	200, 400
Phosphodiesterase-4 inhibitors (PDE4i)	Cilomilast b.i.d.	5, 10, 15
	Roflumilast q.d.	250, 500
Neutrophil elastase inhibitors (NEi)	AZD9668 b.i.d.	60
P38-MAK-kinase inhibitors (P38i)	PH797804 q.d.	0.5, 3, 6, 10
Anticholinergic and β2-Agonist (MABA)	Batefenterol q.d.	37.5, 75, 100, 150, 300, 400, 600, 800
	Batefenterol b.i.d.	100, 200, 400

### Model predictive performance and extension to new drugs

**Fig. 1:**
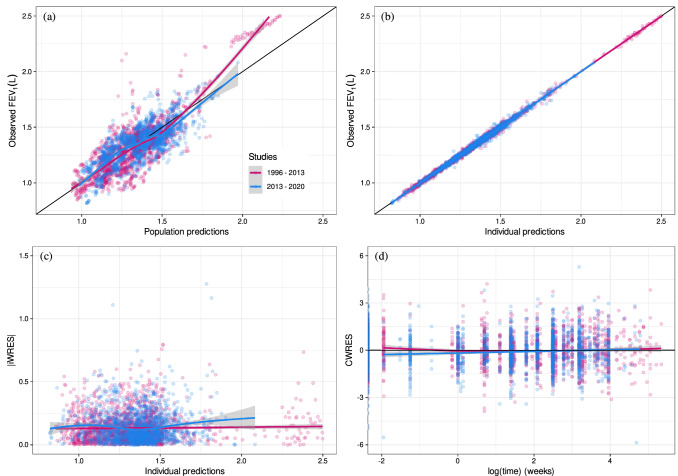
Goodness-of-fit plots for the MBMA published model using the combined dataset and published MBMA estimates **a** Observed FEV_1_ vs. population predictions; **b** Observed FEV_1_ vs. individual predictions; **c** Absolute individual weighted residuals vs. individual predictions; and **d** Conditional weighted residuals vs. time

Goodness-of-fit plots obtained from the legacy MBMA [7] are displayed in Fig. 1. Based on these plots, the current model seems to predict the post-2013 data reasonably well. Therefore, no changes in the structural, statistical and covariate models were performed.

**Fig. 2:**
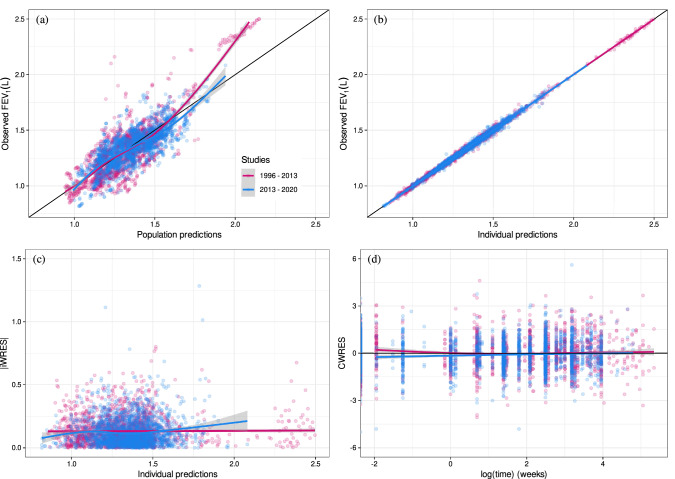
Goodness-of-fit plots for the post-2013 MBMA model using the combined dataset and including new drugs **a** Observed FEV_1_ vs. population predictions; **b** Observed FEV_1_ vs. individual predictions; **c** Absolute individual weighted residuals vs. individual predictions; and **d** Conditional weighted residuals vs. time

Four new compounds (olodaterol, revefenacin, batefenterol and FF) were included in the analysis. New parameter estimates for the augmented data are presented in Table S2 in Supplementary material and the predictive performance of the post-2013 model is shown in Fig. 2.

A correction factor (Eq. 1) with a typical value (relative standard error – RSE%) of 0.89 (0.7) was estimated for six studies accounting for absolute trough FEV_1_ data obtained from CFB with FEV_1_ being measured post-SABD. A positive estimate for θ_correction_ will increase the model-predicted FEV_1_ to appropriately fit the data with augmented absolute FEV_1_ (studies with baseline measurement obtained post-SABD). For example, a predicted transformed absolute FEV_1_ value of 1 L is increased by 0.11 L. As shown in Table 3, the typical estimated efficacy (95%CI) for the reference dose (Eff_ref_) for olodaterol, revefenacin, batefenterol and FF are 89 mL (82.5–96.0), 144 mL (128.4–159.3), 190 mL (165.3–214.8) and 43.6 mL (33.4–53.7), respectively. A typical value (RSE%) of B is 1.17 (1.0) with a coefficient of variation (CV) for ISV and IAV of 10% and 2.1%, respectively for a typical study size of 200 patients. The typical rate for disease progression is 32 mL/per year (RSE: 9.6%) with an ISV CV of 54%. The half-maximum placebo effect was reached after 20 weeks (compared to 11 weeks previously reported) for those studies with gradual onset, whereas 45% of the studies were estimated to have an immediate placebo effect (compared to 48% previously reported).

**Table 3:** Estimates of FEV_1_ effects for all compounds based on an untreated baseline FEV_1_ of 1.2 L

Compound	Reference dose [ug] (dosing frequency)	ED_50_ (95%CI) [ug]	Eff_ref_ (95%CI) [mL]	E_max_ [mL]^a^
Arformoterol	25 (b.i.d.)	0.75 (0.05–12.0)^b^	81.0 (70.1–157.1)^c^	82.3 (71.0–231.1)^d^
Formoterol	9 (b.i.d.)	1.50 (0.09–24.0)	71 (59.0–82.1)	82.3 (71.0–231.1)
Indacaterol	75 (q.d.)	5.37 (0.10–287.4)	128.5 (113.6–143.3)	137.7 (126.0–567.6)
Salmeterol	- (b.i.d.)^e^	**–**	79.3 (70.1–88.4)	**–**
Vilanterol	25 (q.d.)	2.02 (1.67–2.47)	114 (105.4–122.8)	123.4 (114.2–132.4)
Olodaterol	5 (q.d.)	1.47 (0.65–3.33)	89.3 (82.5–96.0)	115.6 (97.7–151.6)
	- (b.i.d.)	**–**	110.6 (105.8–115.3)	**–**
Aclidinium	200 (q.d.)	55.8 (37.4–83.3)	71.2 (55.9–86.5)	91.0 (77.7–103.9)
	400 (b.i.d.)	94.9 (35.4–246.6)	98.2 (83.7–112.6)	121.4 (97.1–167.3)
BEA2180	- (q.d.)	**–**	103.7 (96.9–110.4)	**–**
Glycopyrronium	100 (q.d.)	10.1 (4.61–22.2)	131 (118.3–143.8)	144.3 (124.5–173.4)
GSK233705	- (q.d.)	**–**	192.8 (180.3–205.3)	**–**
Tiotropium – Handihaler (blinded)	18 (q.d.)	2.32 (0.84–6.44)	121.9 (111.6–132.2)	137.6 (126.7–160.7)^f^
Tiotropium – Handihaler (open-label)	18 (q.d.)	2.32 (0.84–6.44)^g^	116 (94.6–139.5)	131 (116.9–159.1)
Tiotropium – Respimat	5 (q.d.)	0.74 (0.51–1.42)^h^	119.7 (111.6–127.8)	137.6 (126.7–160.7)^f^
Umeclidinium	- (q.d.)	**–**	144 (131.9–156.8)	**–**
	- (b.i.d.)	**–**	144 (131.9–156.8)	**–**
Revefenacin	175 (q.d.)	47.6 (23.2–97.8)	143.8 (128.4–159.3)	183 (163.0–216.9)
Beclomethasone	–	**–**	57.7 (31.9–83.5)	**–**
Budesonide	160 (b.i.d.)	169.8 (3.64–7915)	30.7 (8.59–52.8)	63.2 (38.1–53.6)
Fluticasone propionate	- (b.i.d.)	**–**	42.3 (31.8–52.7)	**–**
Fluticasone furoate	100 (q.d.)	11.4 (0.23–561)	43.6 (33.4–53.7)	48.5 (34.6–312.7)
Mometasone	- (q.d.)	**–**	68.8 (54.9–82.7)	**–**
	- (b.i.d.)	**–**	56.3 (43.9–68.6)	**–**
Cilomilast	- (b.i.d.)	**–**	42.7 (33.6–51.8)	**-**
Roflumilast	500 (q.d.)	162.5 (16.7–1579)	78.6 (54.4–102.8)	104.9 (76.1–277.2)
AZD9668	- (q.d.)	**–**	12.2 (0–33.1)	**-**
PH797804	- (q.d.)	**–**	92.5 (70.3–114.6)	**-**
Batefenterol	400 (q.d.)	20.2 (2.93–139.1)	190 (165.3–214.8)	199.6 (166.5–288.3)
	- (b.i.d.)	**–**	207 (196–218.3)	**-**

b.i.d: two times a day

q.d: every day

^a^95%CI of derived E_max_ values were obtained through simulations from the variance-covariance matrix in NONMEM

^b^Constrained to be half of ED_50_ for formoterol

^c^value derived from ED_50_ E_max_ in relation to formoterol. The 95%CI of this value were obtained through simulations from the variance-covariance matrix in NONMEM

^d^constrained to be equal to E_max_ of formoterol

^e^Compounds with missing reference dose indicates that ED_50_ was unidentifiable

^f^E_max_constrained to be the same for both tiotropium blinded and Respimat

^g^constrained to be equal to the ED_50_ of blinded tiotropium

^h^: calculated based on the ED_50_ and E_max_ of blinded tiotropium

**Fig. 3:**
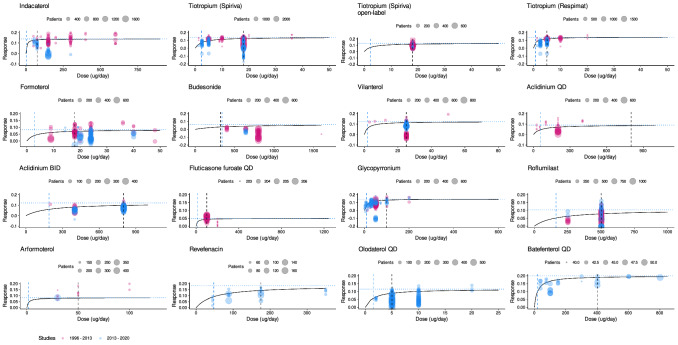
Dose-response relationship for fourteen compounds (given as mono-therapy). Reference doses (black dotted line); estimated ED_50_ (vertical blue dotted line) and E_max_ (horizontal blue dotted line) parameters and FEV_1_ response reported in studies between 1996 and 2013 (pink) and between 2013 and 2020 (blue) are shown

Treatment effect was reduced in more severe patients with a B_i.j_ less than 1.2 L (Eq. 9). Such reduction is directly correlated with the untreated study arm FEV_1_ baseline [7]. For example, a study arm baseline of 1 L resulted in a reduction of 5.4% and 12.8% in the effect of long-acting bronchodilators and anti-inflammatory treatments, respectively. Dose-response relationship was identified for fourteen compounds (Fig. 3). The addition of onset effect was not supported for any of the new drugs included in the analysis, with a difference in OFV of 13 for FF and 0 for both olodaterol and revefenacin.

### MBMA prediction of exacerbation rate

**Fig. 4:**
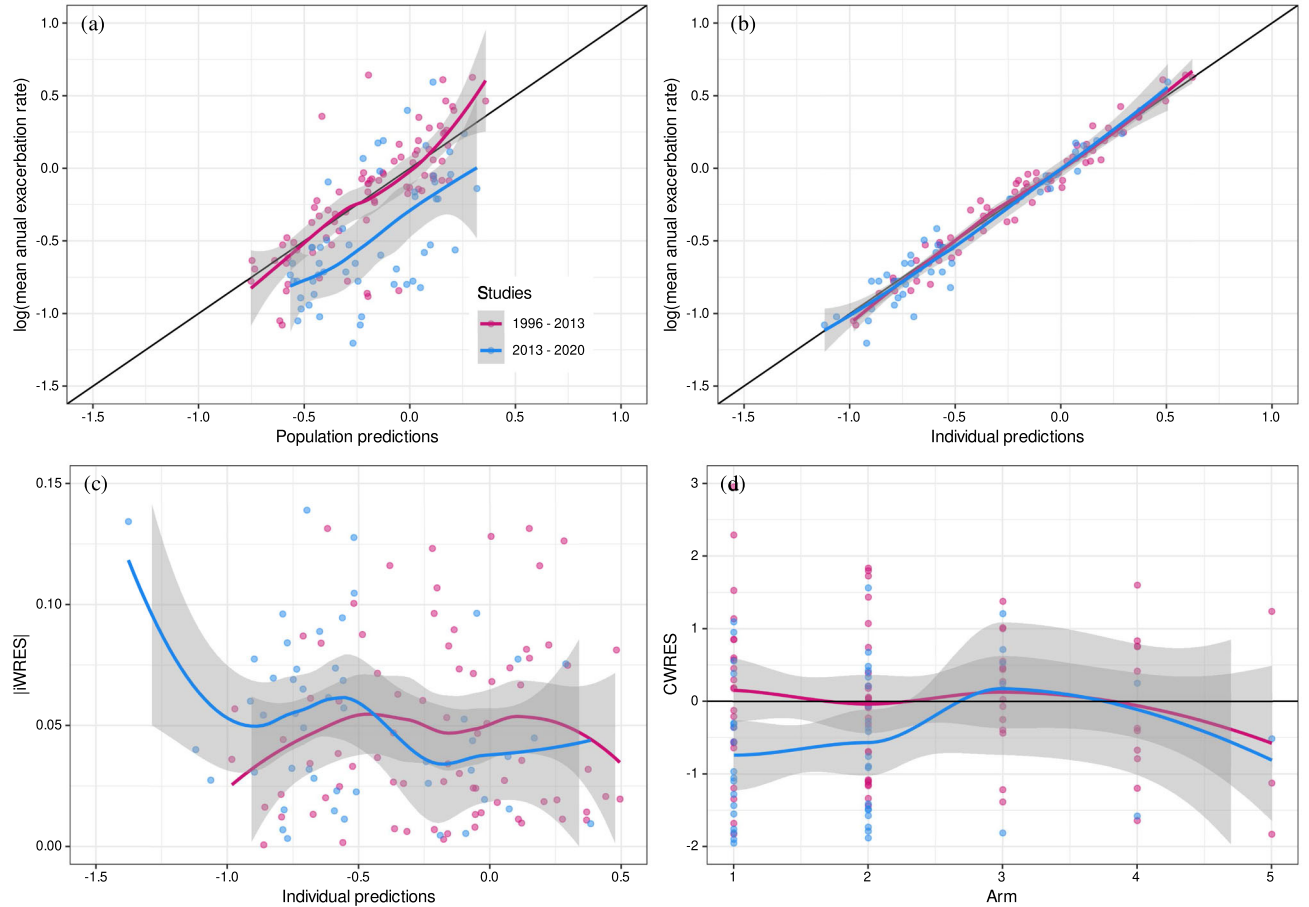
Goodness-of-fit plots for the exacerbation rate model using the combined dataset and published model. **a** Mean annual exacerbation rate vs. population predictions, **b** Mean annual exacerbation rate vs. individual predictions, **c** Absolute individual weighted residuals vs. individual predictions and **d** Conditional weighted residuals vs. arm

Goodness-of-fit plots for the published exacerbation model [10] (parameters shown in Table 4) are displayed in Fig. 4. Based on the observations vs. population predictions, the legacy model overpredicts the post-2013 mean annual exacerbation rate data. Therefore, changes to the model using the augmented data were performed as follows. In the legacy model, different centering values were used for covariates (ppFEV_1_ and ICS_p_) to assess the effect of history of exacerbation (θ_EHIST_) on TVER_PBOi,j_ (Eq. 11); however, this may force θ_EHIST_ to be different than zero. Therefore, in this analysis, the same centering values for covariates were considered. This gave an estimate of -0.12 for θ_EHIST_ compared to -0.30 (case when covariates were centered separately). Additionally, removing θ_EHIST_ did not result in a statistically significant increase in the OFV (1.11 points) meaning that E_HIST_ did not have a meaningful impact in the model. Based on this, the θ_EHIST_ parameter was removed from the model (Eq. 16) and then the covariate effect was tested.

**Table 4:** Parameter estimates for the exacerbation model

Parameter	Estimate (RSE%) [shrinkage SD%]	
	Published study ^a^[10]	New estimates using the post-2013 model^b^
Placebo exacerbation rate (exacerbations per year) for a trial that required a history of exacerbation	1.344 (6.8)	0.909 (7.22)^c^
Fractional change in exacerbation rate for a trial that did not require a history of exacerbation	− 0.328 (20)	–
Fractional change in exacerbation rate for 1% point change in %predicted FEV_1_	− 0.027 (39)	− 0.026 (19.5)
Fractional change in exacerbation rate for 1% point increase in medication history with ICS	0.004 (47)	0.007 (19.2)
Fractional change in exacerbation rate for each unit (L) of drug effect from a bronchodilator or naïve anti-inflammatory effect	− 1.66 (6.7)	− 1.69 (14.7)
Power model for the treatment duration on residual error	− 0.422 (61)	− 0.429 (39.3)
Exponent on L drug effect from an anti-inflammatory in anti-inflammatory experienced patients	0.427 (14)	0.476 (8.36)
Power model for study year as a covariate	–	− 81.0 (16.6)
ISV variance for the placebo exacerbation rate	0.056 (20.9)	0.070 (11.8) [2.32]
Variance for the additive residual error	0.0058 (9.72)	0.0072 (7.94) [20]

^a^Estimates used to generate Fig. 4; ^b^Estimates used to generate Fig. 5; ^c^New estimate does not include history of exacerbation

As an attempt to explain the differences seen between the pre-2013 and post-2013 data sets and thus improve the predictive performance of the model, covariates such as the type of therapy (TTYPE; e.g., mono-, dual- or triple-therapy) and year when the study started (Study_year_) were tested on the pre-treatment placebo rate (TVER_PBOi,j_) as shown in Eq. 17 and Eq. 18, respectively.

16 $${\text{T}\text{V}\text{E}\text{R}}_{\text{P}\text{B}{\text{O}}_{\text{i},\text{j}}}= {{\uptheta }}_{\text{E}\text{R}\text{P}\text{B}\text{O}\text{i},\text{j}}\cdot \text{exp}\left(\right(\text{p}\text{p}{\text{F}\text{E}\text{V}1}_{{1}_{\text{i},\text{j}}}-41)\cdot {{\uptheta }}_{\text{E}\text{R}}+({\text{I}\text{C}\text{S}}_{\text{p}_{\text{i},\text{j}}}-61)\cdot {{\uptheta }}_{\text{H}\text{I}\text{C}\text{S}})$$

17 $${\text{E}\text{R}}_{\text{P}\text{B}{\text{O}}_{\text{i},\text{j}}}=\left\{\begin{array}{c}({\text{T}}{\text{V}}{\text{E}\text{R}}_{\text{P}\text{B}{\text{O}}_{\text{i},\text{j}}}+{{\uptheta }}_{\text{d}\text{u}\text{a}\text{l}})\cdot \text{e}\text{x}\text{p}\left({{\upeta }}_{\text{E}\text{R} }\right) \,If\,\,\,TTYPE=dual\\ ({\text{T}}{\text{V}}{\text{E}\text{R}}_{\text{P}\text{B}{\text{O}}_{\text{i},\text{j}}}+{{\uptheta }}_{\text{t}\text{r}\text{i}\text{p}\text{l}\text{e}})\cdot \text{e}\text{x}\text{p}\left({{\upeta }}_{\text{E}\text{R} }\right) \,If\,\,\,TTYPE=triple\end{array}\right.$$

18 $$\text{E}\text{R}_{\text{P}\text{B}{\text{O}}_{\text{i},\text{j}}}= {\text{T}\text{V}}{\text{E}\text{R}}_{\text{P}\text{B}{\text{O}}_{\text{i},\text{j}}}\cdot {\left(\left.\frac{{\text{S}\text{t}\text{u}\text{d}\text{y}}_{\text{y}\text{e}\text{a}\text{r}}}{2013}\right)\right.}^{{{\uptheta }}_{{\text{S}\text{t}\text{u}\text{d}\text{y}}_{\text{y}\text{e}\text{a}\text{r}}}}\cdot \text{e}\text{x}\text{p}\left({{\upeta }}_{\text{E}\text{R} }\right)$$

**Fig. 5:**
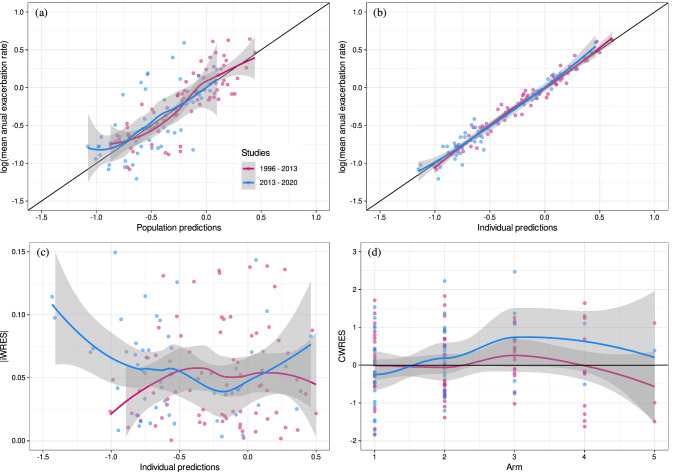
Goodness-of-fit plots for the post-2013 exacerbation rate model using the combined dataset. **a** Mean annual exacerbation rate vs. population predictions, **b** Mean annual exacerbation rate vs. individual predictions, **c** Absolute individual weighted residuals vs. individual predictions and **d** Conditional weighted residuals vs. arms

Inclusion of TTYPE and Study_year_ resulted in a decrease in OFV of 4 and 23 points, respectively. Model parameter estimates and goodness-of-fit-plots for the post-2013 model including Study_year_ are shown in Table 4 and Fig. 5, respectively. A typical value (RSE%) of placebo exacerbation rate is 0.91 (7.22) with a CV for ISV of 27%.

**Fig. 6:**
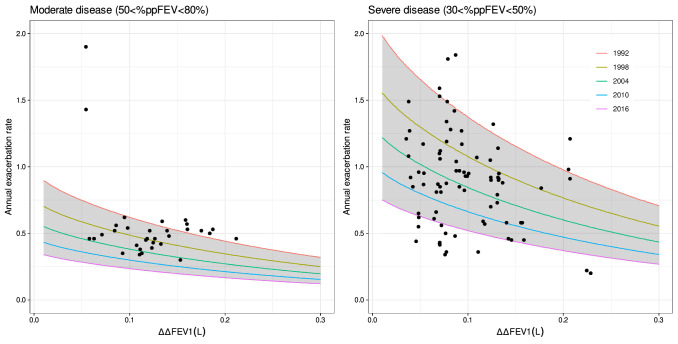
Relationship between predicted mean annual exacerbation rate and placebo adjusted drug effect on FEV_1_ for different study year in patients with moderate and severe disease. For predictions, it was assumed a predicted %FEV1 (ppFEV_1_) of 70 and 40 for moderate and severe patients, respectively; and 61% of patients required to wash out from ICS (Eq. 16). Dots are observed data for moderate (left panel) and severe (right panel) patients; lines are the median annual exacerbation rate for a given year; ribbon represents the continuous interval for studies from 1992 and 2016; ΔΔFEV_1_ is the placebo adjusted change from baseline in FEV_1_. ISV and IAV are not included in the predictions

The relationship between predicted mean annual exacerbation rate and ΔΔFEV_1_ for moderate and severe disease at different years is displayed in Fig. 6. In a typical study from 2016 with 61% of patients having a history of ICS usage, the model predicted that a reduction in exacerbation rate of at least 20% can be achieved with a reduction in ΔΔFEV_1_ of at least 47 mL in moderate and severe patients (Fig. 6).

### Comparative effectiveness

**Fig. 7:**
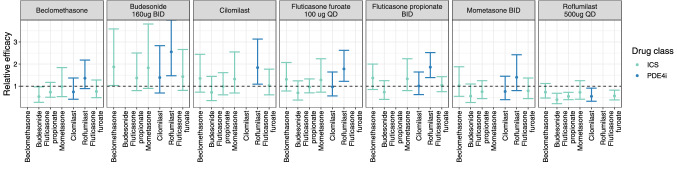
Comparative effectiveness of anti-inflammatory drugs. Point estimate and 95% CI for the relative obtained using the llp option in PsN is displayed for all drugs. Plot is stratified by drug 2

**Fig. 8:**
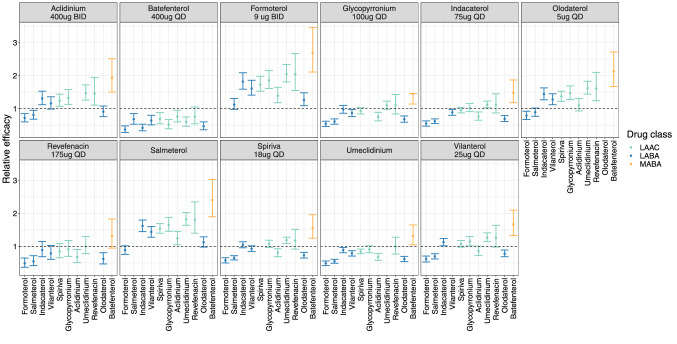
Comparative effectiveness of bronchodilator drugs. Point estimate and 95% CI for the relative obtained using the llp option in PsN is displayed for all drugs. Plot is stratified by drug 2

The relative effect for anti-inflammatory drugs and bronchodilators using each drug as a reference is shown in Figs. 7 and 8, respectively. For most anti-inflammatories, superiority or inferiority cannot be established. However, roflumilast is superior to most of the drugs except beclomethasone and mometasone. In case of bronchodilators, batefenterol shows to be superior to most of the drugs except revefenacin where superiority/inferiority cannot be established (Fig. 8). Furthermore, umeclidinium shows to be superior to all bronchodilators except batefenterol (which shows to be superior), and revefenacin and glycopyrronium where superiority/inferiority cannot be established. Vilanterol is superior to formoterol, salmeterol and olodaterol only.

## Discussion

This study assessed the predictive performance of a model-based longitudinal meta-analysis for FEV_1_ [7] in patients with COPD following inhaled treatment with anti-inflammatories and bronchodilators and its link to mean annual exacerbation rate [10] using augmented data with additional RCTs published between 2013 and 2020. This resulted in at least doubling the number of observations with more than 2000 additional morning trough FEV_1_ (data from 134960 randomized patients in 156 studies) for analysis, and showed that the legacy model [7] can predict these data well. Furthermore, the model was updated incorporating additional drugs as well as drug combinations with new estimates of FEV_1_ effects. The parameter estimates based on the augmented data (post-2013 model) are generally in line with the legacy MBMA estimates of drugs in the same class (Table S2 in supplementary material). Although the population model predictions adequately described the observed data (Fig. 2a), the post-2013 model cannot reliably predict at the individual level, as evidenced by the shrinkage (standard deviation%) for the residual error is 20% for the FEV_1_ vs. individual prediction plot. To some extent, this can be explained by the sparse data per study for the MBMA (median [range] observations per study arm of 4 [1–13]).

Notably, the typical new estimated efficacy of the reference dose for tiotropium (Respimat) was lower compared to previous estimates (120 mL vs. 134 mL) as well as for aclidinium b.i.d. (98 mL vs. 120 mL) and vilanterol (114 mL vs. 139 mL). This could be explained by the infra-additive LABA + LAAC interaction considered in the model resulting in a reduced efficacy of long-acting bronchodilators [19], and the higher percentage of patients receiving LABA and LAAC as a background medication during the study period in the post-2013 data (Table 1). On another note, the post-2013 model predicts a reduction of 5.4% in the effect of bronchodilator treatment for more severe patients (study arm baseline FEV_1_ < 1.2 L), in line with the legacy MBMA (4.7%). However, a lower reduction in the effect of anti-inflammatory treatments was estimated in the post-2013 model (12.5% vs. 28%).

In the post-2013 model, the typical rate of disease progression is 32 mL/year which is in line with previous report (33 mL/year) [20]. A faster disease progression has been observed in current smokers compared to former smokers (mean rate of decline in FEV_1_ of at least 21 mL/year greater in smokers) [20]. However, smoking status was not identified as a significant covariate on disease progression. This could be due to the nature of aggregate covariate data that describe a narrower range of values than individual covariate data making the covariate analysis difficult. Additionally, longer studies might be required to capture disease progression as has been previously pointed out [7]. Some benefits of combining individual patient level data and aggregate data for analysis include to (i) characterize patient-level relationships; (ii) better describe the effect of covariates, and the correlation among outcomes; and (iii) make the model suitable for predictions or simulations of individual outcomes.

Dose-response relationship was identified for fourteen out of twenty-three compounds (Fig. 3). For indacaterol, the new estimate of the typical value of ED_50_ is significantly smaller (with a larger uncertainty) compared to the published estimate (5.37 ug vs. 24.3 ug) (Table S2 in supplementary material). For this drug, it has been shown that baseline FEV_1_, as a marker of disease severity, influences the dose-response with less severe COPD patients requiring lower doses to achieve optimal effect [21]. For study treatment arms allocated to indacaterol, there is a mean difference of 13 mL in FEV_1_ at baseline between pre-2013 (mean FEV_1_ of 1.351 L) and post-2013 dataset (mean FEV_1_ of 1.338 L) meaning that in the post-2013 data, patients receiving indacaterol had a slightly less severe COPD than patients in the pre-2013 data. However, such difference might not deem to be clinically relevant. Usually, large phase 3 and 4 studies assess a clinical dose instead of a wide range of doses which may contribute to the relatively high uncertainty seen in the ED_50_ parameter estimates for some drugs. The dose-response for umeclidinium has been already characterized [22]; however, in this study, the ED_50_ parameter for umeclidinium was unidentifiable possibly due to the lack of data at the lower dose range (only one study with approximately 150 patients in total [22]).

In this analysis, the inclusion of onset of effect for the new drugs was not supported. Bronchodilators such as olodaterol and revefenacin have a fast onset of action (5 and 45 min post-dose for olodaterol [23] and revefenacin [24], respectively). To describe their effect onset, earlier FEV_1_ time points would have been required.

Comparative effectiveness is an important decision-making component during clinical drug development. As shown in this analysis as well as previously [17], MBMA is a useful tool to leverage use of the available data efficiently, and compare efficacy across different drugs even in the absence of real-life head-to-head trials. A comparative effectiveness analysis was performed using the post-2013 MBMA model as described before [17]. Roflumilast, a PDE4 inhibitor, showed to be superior to most of the other anti-inflammatories based on pulmonary response except for beclomethasone and mometasone where superiority/inferiority could not be determined. A comparison between inhaled beclomethasone and roflumilast in patients with persistent asthma showed a comparable effect between the two drugs in improving pulmonary function [25]. Superiority or inferiority of the inhaled ICS, FF, relative to most of anti-inflammatories could not be established when given as a mono-therapy. Differences among bronchodilators are more evident compared to anti-inflammatory drugs. Formoterol and salmeterol showed to be inferior to most of the other long acting bronchodilators, which is in agreement with the previous analysis [17].

Different bronchodilators and combinations have been compared in head-to-head trials. For example, umeclidinium showed to be non-inferior to once-daily glycopyrronium but superior to tiotropium in patients with COPD based on trough FEV_1_ at day 85 [26, 27]. This is in line with our findings as shown in Fig. 8. Regarding olodaterol, in this analysis, it showed to be inferior to most of other bronchodilators. A direct comparison between tiotropium/olodaterol and umeclidinium/vilanterol, superiority with umeclidinium/vilanterol was observed for the primary end point of trough FEV_1_ at week 8 [28]; however, non-inferiority could be established when compared umeclidinium/vilanterol with indacaterol/glycopyrronium (endpoint of trough FEV_1_ at week 12) [29]. Comparative effectiveness between different combinations was not assessed in this study.

The exacerbation model used in this analysis [10] predicts a higher exacerbation rate as a function of higher ppFEV_1_ as well as higher percentage of patients receiving ICS prior to randomization, both indicative of COPD disease severity. The same relationship between disease severity (ppFEV1), use of ICS prior randomization and exacerbation rate was shown in a model that describes exacerbation rates as a function of treatment duration in patients receiving roflumilast [30]. Currently, history of exacerbations remains the most important predictor of future exacerbations [31]. The need to be treated with ICS prior to randomization may indicate a previous exacerbation history. This potential correlation between use of ICS and an exacerbation event could explain why the model did not support the addition of exacerbation history as a covariate.

The published estimates for the exacerbation model [10] (Table 4) tends to overpredict the post-2013 mean annual exacerbation rate data. The inclusion of study year on baseline was the only statistically significant covariate. This covariate effect may be explained by (i) an improvement in the disease management over time and/or (ii) an increase in the number of marketed therapies which include dual and triple combinations (14% and 6% more studies compared to pre-2013 data, respectively). However, the authors acknowledge that study year might be a confounding factor, masking other plausible explanations for the differences in the pre-2013 and post-2013 data (i.e., measurement error). Furthermore, study year would not capture significant changes in exacerbation rate if simulations for future comparison of drugs are performed (e.g., exacerbation rate may be similar between 2016 and 2020). Future studies focusing on assessment of other potential predictors of exacerbation, for instance dyspnea, cough and sputum, exercise capacity and/or peripheral eosinophil count (PBE) may provide additional insight. This seems to be important considering the worse survival outcome associated with severe or frequent exacerbations [32]. Current evidence suggests the use of PBE when deciding on ICS use [9]; however, the link between PBE counts with future exacerbation risk is inconclusive and even controversial in COPD. In this study, only 6% of the trials that reported annual exacerbation rate had PBE data, and therefore it was not tested in the model.

This MBMA could serve as a tool to make quantitative decisions during drug development, specifically for trial design selection and optimization. For instance, clinical trial simulations can help to improve our understanding on first-in-patient trial design performance to inform Phase 2 decision making [33, 34], or assess the probability of detecting drug effect as a function of sample size [35]. Applications of MBMA to support drug development decision-making have been already pointed out [36]. Furthermore, the influence of varying inclusion criteria on the trial outcome in terms of effect size and power can also be quantified. This can be of interest in light of the stringent inclusion criteria for RCTs, which makes study findings less generalizable to a broader population [37].

## Conclusion

The addition of 7 years’ worth of new data to the legacy COPD MBMA enabled a more robust model with increased predictability performance. This study shows that the legacy MBMA model is consistent and can be considered as a general model to predict FEV_1_ when using bronchodilators and anti-inflammatories in COPD. Furthermore, the comparison effectiveness analysis performed in this study demonstrated the usefulness of the MBMA, with results, where available, being in line with real-life head-to-head trials. An updated exacerbation model has been presented where study year seems to be an important covariate to explain potential improvements in the disease management over time. Future studies should focus on integrating aggregate and individual level patient data into the MBMA to make the analysis suitable for individual outcomes predictions.

## Supplementary Information

Below is the link to the electronic supplementary material.

Supplementary material 1 (PDF 136 kb)
