# Supplementary material for: Joint longitudinal model-based meta-analysis of FEV1 and exacerbation rate in randomized COPD trials
Source: J Pharmacokinet Pharmacodyn. 2023 Mar 22;50(4):297–314. doi: 10.1007/s10928-023-09853-z (PMC10374752; doi:10.1007/s10928-023-09853-z)
Supplement: Supplementary file 1 — Supplementary material 1 (PDF 136 kb) [file 10928_2023_9853_MOESM1_ESM.pdf]

Supplementary material for article **"Joint longitudinal model-based meta-analysis of FEV_1 and exacerbation rate in randomized COPD trials"**

Carolina Llanos-Paez[1], Claire Ambery[2], Shuying Yang[2], Misba Beerahee[2], Elodie L. Plan[1], Mats O. Karlsson[1*]

[1] Department of Pharmacy, Uppsala University, Uppsala, Sweden.
[2] Clinical Pharmacology Modelling and Simulation, GSK, London, UK.

***Corresponding author:** Mats O. Karlsson, Department of Pharmacy, Uppsala University, BMC, Box 580 751 23 Uppsala, Sweden; +4618-471 4105; mats.karlsson@farmaci.uu.se

Table S1. References (n=132) from 2013 to 2020 included in the analysis comprising 156 studies

Reference	Number of subjects	Treatment duration (weeks)	Study code
Siler TM, Moran EJ, Barnes CN, Crater GD. Safety and Efficacy of Revefenacin and Formoterol in Sequence and Combination via a Standard Jet Nebulizer in Patients with Chronic Obstructive Pulmonary Disease: A Phase 3b, Randomized, 42-Day Study. Chronic Obstr Pulm Dis. 2020 Apr;7(2):99-106. doi: 10.15326/jcopdf.7.2.2019.0154.	122	6	NCT03573817
Ferguson GT, Brown N, Compton C, Corbridge TC, Dorais K, Fogarty C, Harvey C, Kaisermann MC, Lipson DA, Martin N, Sciurba F, Stiegler M, Zhu CQ, Bernstein D. Once-daily single-inhaler versus twice-daily multiple-inhaler triple therapy in patients with COPD: lung function and health status results from two replicate randomized controlled trials. Respir Res. 2020 May	720 + 711	12	NCT03478683<br>NCT03478696
Crim C, Gotfried M, Spangenthal S, Watkins M, Emmett A, Crawford C, Baidoo C, Castro-Santamaria R. A randomized, controlled, repeat-dose study of batefenterol/fluticasone furoate compared with placebo in the treatment of COPD. BMC Pulm Med. 2020 May 4;20(1):119. doi: 10.1186/s12890-020-1153-7.	62	6	NCT02573870
Mahler DA, Ohar JA, Barnes CN, Moran EJ, Pendyala S, Crater GD. Nebulized Versus Dry Powder Long-Acting Muscarinic Antagonist Bronchodilators in Patients With COPD and Suboptimal Peak Inspiratory Flow Rate. Chronic Obstr Pulm Dis. 2019 Oct 23;6(4):321–31. doi: 10.15326/jcopdf.6.4.2019.0137.	206	4	NCT03095456
Maltais F, Hamilton A, Voß F, Maleki-Yazdi MR. Dose Determination for a Fixed-Dose Drug Combination: A Phase II Randomized Controlled Trial for Tiotropium/Olodaterol Versus Tiotropium in Patients with COPD. Adv Ther. 2019 Apr;36(4):962-968. doi: 10.1007/s12325-019-00911-y.	537	4	NCT00696020
Maltais F, Bjermer L, Kerwin EM, Jones PW, Watkins ML, Tombs L, Naya IP, Boucot IH, Lipson DA, Compton C, Vahdati-Bolouri M, Vogelmeier CF. Efficacy of umeclidinium/vilanterol versus umeclidinium and salmeterol monotherapies in symptomatic patients with COPD not receiving inhaled corticosteroids: the EMAX randomised trial. Respir Res. 2019 Oct 30;20(1):238. doi: 10.1186/s12931-019-1193-9	2,431	24	NCT03034915
Sethi S, Kerwin E, Watz H, Ferguson GT, Mroz RM, Segarra R, Molins E, Jarreta D, Garcia Gil E. AMPLIFY: a randomized, Phase III study evaluating the efficacy and safety of aclidinium/formoterol vs monocomponents and tiotropium in patients with moderate-to-very severe symptomatic COPD. Int J Chron Obstruct Pulmon Dis. 2019 Mar 22;14:667-682. doi: 10.2147/COPD.S189138	1,594	24	NCT02796677
Crim C, Watkins ML, Bateman ED, Feldman GJ, Schenkenberger I, Kerwin EM, Crawford C, Pudi K, Ho S, Baidoo C, Castro-Santamaria R. Randomized dose-finding study of batefenterol via dry powder inhaler in patients with COPD. Int J Chron Obstruct Pulmon Dis. 2019 Mar 8;14:615-629. doi: 10.2147/COPD.S190603	323	6	NCT02570165

Lehmann S, Ringbæk T, Løkke A, Grote L, Hedner J, Lindberg E. A randomized trial to determine the impact of indacaterol/glycopyrronium on nighttime oxygenation and symptoms in patients with moderate-to-severe COPD: the DuoSleep study. Int J Chron Obstruct Pulmon Dis. 2019 Jan 9;14:199-210. doi: 10.2147/COPD.S184127	38	4	NCT02233543
Hanania NA, Sethi S, Koltun A, Ward JK, Spanton J, Ng D. Long-term safety and efficacy of formoterol fumarate inhalation solution in patients with moderate-to-severe COPD. Int J Chron Obstruct Pulmon Dis. 2018 Dec 27;14:117-127. doi: 10.2147/COPD.S173595.	1,071	52	NCT01488019
Riley JH, Kalberg CJ, Donald A, Lipson DA, Shoaib M, Tombs L. Effects of umeclidinium/vilanterol on exercise endurance in COPD: a randomised study. ERJ Open Res. 2018 Jan 5;4(1):00073-2017. doi: 10.1183/23120541.00073-2017	198	12	NCT02275052
Ferguson GT, Papi A, Anzueto A, Kerwin EM, Cappelletti C, Duncan EA, Nyberg J, Dorinsky P. Budesonide/formoterol MDI with co-suspension delivery technology in COPD: the TELOS study. Eur Respir J. 2018 Sep 16;52(3):1801334. doi: 10.1183/13993003.01334-2018.	2,389	24	NCT02766608
Beeh KM, Kirsten AM, Tanase AM, Richard A, Cao W, Hederer B, Beier J, Kornmann O, van Zyl-Smit RN. Indacaterol acetate/mometasone furoate provides sustained improvements in lung function compared with salmeterol xinafoate/fluticasone propionate in patients with moderate-to-very-severe COPD: results from a Phase II randomized, double-blind 12-week study. Int J Chron Obstruct Pulmon Dis. 2018 Dec 6;13:3923-3936. doi: 10.2147/COPD.S179293	629	12	NCT01636076
Rabe KF, Watz H, Baraldo S, Pedersen F, Biondini D, Bagul N, Hanauer G, Göhring UM, Purkayastha D, Román J, Alagappan VKT, Saetta M. Anti-inflammatory effects of roflumilast in chronic obstructive pulmonary disease (ROBERT): a 16-week, randomised, placebo-controlled trial. Lancet Respir Med. 2018 Nov;6(11):827-836. doi: 10.1016/S2213-2600(18)30331-X	158	16	NCT01509677
Reisner C, Pearle J, Kerwin EM, Rose ES, Darken P. Efficacy and safety of four doses of glycopyrrolate/formoterol fumarate delivered via a metered dose inhaler compared with the monocomponents in patients with moderate-to-severe COPD. Int J Chron Obstruct Pulmon Dis. 2018 Jun 19;13:1965-1977. doi: 10.2147/COPD.S166455	185	1	NCT01349816
Kerwin EM, Siler TM, Arora S, Darken P, Rose E St, Reisner C. Efficacy, safety, and pharmacokinetics of budesonide/formoterol fumarate delivered via metered dose inhaler using innovative co-suspension delivery technology in patients with moderate-to-severe COPD. Int J Chron Obstruct Pulmon Dis. 2018 May 8;13:1483-1494. doi: 10.2147/COPD.S164281	180	4	NCT02196077
Fukushima Y, Nakatani Y, Ide Y, Sekino H, St Rose E, Siddiqui S, Maes A, Reisner C. Randomized, double-blind, placebo-controlled trial to assess the efficacy and safety of three doses of co-suspension delivery technology glycopyrronium MDI in Japanese patients with moderate-to-severe COPD. Int J Chron Obstruct Pulmon Dis. 2018 Apr 13;13:1187-1194. doi: 10.2147/COPD.S159246	66	1	NCT03256552

Kerwin EM, Spangenthal S, Kollar C, St Rose E, Reisner C. A phase IIb randomized, chronic-dosing, incomplete block, cross-over study of glycopyrronium, delivered via metered dose inhaler, compared with a placebo and an active control in patients with moderate-to-severe COPD. Respir Res. 2018 Mar 5;19(1):38. doi: 10.1186/s12931-018-0739-6	103	1	NCT01350128
Hohlfeld JM, Vogel-Claussen J, Biller H, Berliner D, Berschneider K, Tillmann HC, Hiltl S, Bauersachs J, Welte T. Effect of lung deflation with indacaterol plus glycopyrronium on ventricular filling in patients with hyperinflation and COPD (CLAIM): a double-blind, randomised, crossover, placebo-controlled, single-centre trial. Lancet Respir Med. 2018 May;6(5):368-378. doi: 10.1016/S2213-2600(18)30054-7	62	2	NCT02442206
Papi A, Vestbo J, Fabbri L, Corradi M, Prunier H, Cohuet G, Guasconi A, Montagna I, Vezzoli S, Petruzzelli S, Scuri M, Roche N, Singh D. Extrafine inhaled triple therapy versus dual bronchodilator therapy in chronic obstructive pulmonary disease (TRIBUTE): a double-blind, parallel group, randomised controlled trial. Lancet. 2018 Mar 17;391(10125):1076-1084. doi: 10.1016/S0140-6736(18)30206-X	1,532	52	NCT02579850
Bremner PR, Birk R, Brealey N, Ismaila AS, Zhu CQ, Lipson DA. Single-inhaler fluticasone furoate/umeclidinium/vilanterol versus fluticasone furoate/vilanterol plus umeclidinium using two inhalers for chronic obstructive pulmonary disease: a randomized non-inferiority study. Respir Res. 2018 Jan 25;19(1):19. doi: 10.1186/s12931-018-0724-0	1,055	24	NCT02729051
Ferguson GT, Tashkin DP, Skärby T, Jorup C, Sandin K, Greenwood M, Pemberton K, Trudo F. Effect of budesonide/formoterol pressurized metered-dose inhaler on exacerbations versus formoterol in chronic obstructive pulmonary disease: The 6-month, randomized RISE (Revealing the Impact of Symbicort in reducing Exacerbations in COPD) study. Respir Med. 2017 Nov;132:31-41. doi: 10.1016/j.rmed.2017.09.002	1,219	26	NCT02157935
D'Urzo A, Rennard S, Kerwin E, Donohue JF, Lei A, Molins E, Leselbaum A. A randomised double-blind, placebo-controlled, long-term extension study of the efficacy, safety and tolerability of fixed-dose combinations of aclidinium/formoterol or monotherapy in the treatment of chronic obstructive pulmonary disease. Respir Med. 2017 Apr;125:39-48. doi: 10.1016/j.rmed.2017.02.008	921	52	NCT01572792
O'Donnell DE, Casaburi R, Frith P, Kirsten A, De Sousa D, Hamilton A, Xue W, Maltais F. Effects of combined tiotropium/olodaterol on inspiratory capacity and exercise endurance in COPD. Eur Respir J. 2017 Apr 19;49(4):1601348. doi: 10.1183/13993003.01348-2016	295 + 291	6	NCT01533922 NCT01533935
Ichinose M, Kato M, Takizawa A, Sakamoto W, Grönke L, Tetzlaff K, Fukuchi Y. Long-term safety and efficacy of combined tiotropium and olodaterol in Japanese patients with chronic obstructive pulmonary disease. Respir Investig. 2017 Mar;55(2):121-129. doi: 10.1016/j.resinv.2016.09.004.	122	52	NCT01536262
Vestbo J, Papi A, Corradi M, Blazhko V, Montagna I, Francisco C, Cohuet G, Vezzoli S, Scuri M, Singh D. Single inhaler extrafine triple therapy versus long-acting muscarinic antagonist therapy for chronic obstructive pulmonary disease (TRINITY): a double-blind, parallel group, randomised controlled trial. Lancet. 2017 May 13;389(10082):1919-1929. doi: 10.1016/S0140-6736(17)30188-5	2,691	52	NCT01911364

Kerwin EM, Kalberg CJ, Galkin DV, Zhu CQ, Church A, Riley JH, Fahy WA. Umeclidinium/vilanterol as step-up therapy from tiotropium in patients with moderate COPD: a randomized, parallel-group, 12-week study. Int J Chron Obstruct Pulmon Dis. 2017 Feb 24;12:745-755. doi: 10.2147/COPD.S119032.	494	12	NCT01899742
Bhatt SP, Dransfield MT, Cockcroft JR, Wang-Jairaj J, Midwinter DA, Rubin DB, Scott-Wilson CA, Crim C. A randomized trial of once-daily fluticasone furoate/vilanterol or vilanterol versus placebo to determine effects on arterial stiffness in COPD. Int J Chron Obstruct Pulmon Dis. 2017 Jan 19;12:351-365. doi: 10.2147/COPD.S117373	430	24	NCT01336608
Siler TM, Nagai A, Scott-Wilson CA, Midwinter DA, Crim C. A randomised, phase III trial of once-daily fluticasone furoate/vilanterol 100/25 µg versus once-daily vilanterol 25 µg to evaluate the contribution on lung function of fluticasone furoate in the combination in patients with COPD. Respir Med. 2017 Feb;123:8-17. doi: 10.1016/j.rmed.2016.12.001	1,620	12	NCT02105974
Chan R, Sousa AR, Hynds P, Homayoun-Valiani F, Edwards D, Tabberer M. Assessment of the efficacy and safety of fluticasone propionate and salmeterol delivered as a combination dry powder via a capsule-based inhaler versus a multi-dose inhaler in patients with chronic obstructive pulmonary disease. Pulm Pharmacol Ther. 2017 Apr;43:12-19. doi: 10.1016/j.pupt.2017.01.009	426	12	NCT01978145
Lipson DA, Barnacle H, Birk R, Brealey N, Locantore N, Lomas DA, Ludwig-Sengpiel A, Mohindra R, Tabberer M, Zhu CQ, Pascoe SJ. FULFIL Trial: Once-Daily Triple Therapy for Patients with Chronic Obstructive Pulmonary Disease. Am J Respir Crit Care Med. 2017 Aug 15;196(4):438-446. doi: 10.1164/rccm.201703-0449OC	1,810	24	NCT02345161
Papi A, Dokic D, Tzimas W, Mészáros I, Olech-Cudzik A, Koroknai Z, McAulay K, Mersmann S, Dalvi PS, Overend T. Fluticasone propionate/formoterol for COPD management: a randomized controlled trial. Int J Chron Obstruct Pulmon Dis. 2017 Jul 5;12:1961-1971. doi: 10.2147/COPD.S136527	1,765	52	2012–004162–17
Kerwin E, Ferguson GT, Sanjar S, Goodin T, Yadao A, Fogel R, Maitra S, Sen B, Ayers T, Banerji D. Dual Bronchodilation with Indacaterol Maleate/Glycopyrronium Bromide Compared with Umeclidinium Bromide/Vilanterol in Patients with Moderate-to-Severe COPD: Results from Two Randomized, Controlled, Cross-over Studies. Lung. 2017 Dec;195(6):739-747. doi: 10.1007/s00408-017-0055-9	357 + 355	12	NCT02487446<br>NCT02487498
Reisner C, Fabbri LM, Kerwin EM, Fogarty C, Spangenthal S, Rabe KF, Ferguson GT, Martinez FJ, Donohue JF, Darken P, St Rose E, Orevillo C, Strom S, Fischer T, Golden M, Dwivedi S. A randomized, seven-day study to assess the efficacy and safety of a glycopyrrolate/formoterol fumarate fixed-dose combination metered dose inhaler using novel Co-Suspension™ Delivery Technology in patients with moderate-to-very severe chronic obstructive pulmonary disease. Respir Res. 2017 Jan 6;18(1):8. doi: 10.1186/s12931-016-0491-8	122	1	NCT01085045
Kerwin E, Donohue JF, Goodin T, Tosiello R, Wheeler A, Ferguson GT. Efficacy and safety of glycopyrrolate/eFlow® CS (nebulized glycopyrrolate) in moderate-to-very-severe COPD: Results from the glycopyrrolate for obstructive lung disease via electronic nebulizer (GOLDEN) 3 and 4 randomized	653 + 641	12	NCT02347761<br>NCT02347774

controlled trials. Respir Med. 2017 Nov;132:238-250. doi: 10.1016/j.rmed.2017.07.011			
Ferguson GT, Taylor AF, Thach C, Wang Q, Schubert-Tennigkeit AA, Patalano F, Banerji D. Long-Term Maintenance Bronchodilation With Indacaterol/Glycopyrrolate Versus Indacaterol in Moderate-to-Severe COPD Patients: The FLIGHT 3 Study. Chronic Obstr Pulm Dis. 2016 Aug 18;3(4):716-728. doi: 10.15326/jcopdf.3.4.2016.0131	615	52	NCT01682863
Kerwin E, Siler TM, Korenblat P, White A, Eckert JH, Henley M, Patalano F, D'Andrea P. Efficacy and Safety of Twice-Daily Glycopyrrolate Versus Placebo in Patients With COPD: The GEM2 Study. Chronic Obstr Pulm Dis. 2016 Mar 28;3(2):549-559. doi: 10.15326/jcopdf.3.2.2015.0157	432	12	NCT01715298
Siler TM, Kerwin E, Singletary K, Brooks J, Church A. Efficacy and Safety of Umeclidinium Added to Fluticasone Propionate/Salmeterol in Patients with COPD: Results of Two Randomized, Double-Blind Studies. COPD. 2016;13(1):1-10. doi: 10.3109/15412555.2015.1034256	617 + 608	12	NCT01772134<br>NCT01772147
Singh D, Papi A, Corradi M, Pavlišová I, Montagna I, Francisco C, Cohuet G, Vezzoli S, Scuri M, Vestbo J. Single inhaler triple therapy versus inhaled corticosteroid plus long-acting β2-agonist therapy for chronic obstructive pulmonary disease (TRILOGY): a double-blind, parallel group, randomised controlled trial. Lancet. 2016 Sep 3;388(10048):963-73. doi: 10.1016/S0140-6736(16)31354-X	1,368	52	NCT01917331
Fabbri LM, Kerwin EM, Spangenthal S, Ferguson GT, Rodriguez-Roisin R, Pearle J, Sethi S, Orevillo C, Darken P, St Rose E, Fischer T, Golden M, Dwivedi S, Reisner C. Dose-response to inhaled glycopyrrolate delivered with a novel Co-Suspension™ Delivery Technology metered dose inhaler (MDI) in patients with moderate-to-severe COPD. Respir Res. 2016 Sep 2;17(1):109. doi: 10.1186/s12931-016-0426-4	140	2	NCT01566773
Martinez FJ, Rabe KF, Sethi S, Pizzichini E, McIvor A, Anzueto A, Alagappan VK, Siddiqui S, Rekeda L, Miller CJ, Zetterstrand S, Reisner C, Rennard SI. Effect of Roflumilast and Inhaled Corticosteroid/Long-Acting β2-Agonist on Chronic Obstructive Pulmonary Disease Exacerbations (RE(2)SPOND). A Randomized Clinical Trial. Am J Respir Crit Care Med. 2016 Sep 1;194(5):559-67. doi: 10.1164/rccm.201607-1349OC	2,354	52	NCT01443845
Vogelmeier C, Paggiaro PL, Dorca J, Sliwinski P, Mallet M, Kirsten AM, Beier J, Seoane B, Segarra RM, Leselbaum A. Efficacy and safety of aclidinium/formoterol versus salmeterol/fluticasone: a phase 3 COPD study. Eur Respir J. 2016 Oct;48(4):1030-1039. doi: 10.1183/13993003.00216-2016	933	24	NCT01908140
Maltais F, Kirsten AM, Hamilton A, De Sousa D, Voß F, Decramer M. Evaluation of the effects of olodaterol on exercise endurance in patients with chronic obstructive pulmonary disease: results from two 6-week crossover studies. Respir Res. 2016 Jul 6;17(1):77. doi: 10.1186/s12931-016-0389-5	151 + 157	6	NCT01040130<br>NCT01040793
LaForce C, Feldman G, Spangenthal S, Eckert JH, Henley M, Patalano F, D'Andrea P. Efficacy and safety of twice-daily glycopyrrolate in patients with stable, symptomatic COPD with moderate-to-severe airflow limitation: the GEM1 study. Int J Chron Obstruct Pulmon Dis. 2016 Jun 8;11:1233-43. doi: 10.2147/COPD.S100445	441	12	NCT01709864

Sousa AR, Riley JH, Church A, Zhu CQ, Punekar YS, Fahy WA. The effect of umeclidinium added to inhaled corticosteroid/long-acting β2-agonist in patients with symptomatic COPD: a randomised, double-blind, parallel-group study. NPJ Prim Care Respir Med. 2016 Jun 23;26:16031. doi: 10.1038/npjpcrm.2016.31	236	12	NCT02257372
Watz H, Mailänder C, Baier M, Kirsten A. Effects of indacaterol/glycopyrronium (QVA149) on lung hyperinflation and physical activity in patients with moderate to severe COPD: a randomised, placebo-controlled, crossover study (The MOVE Study). BMC Pulm Med. 2016 Jun 14;16(1):95. doi: 10.1186/s12890-016-0256-7	194	3	NCT01996319
Donohue JF, Soong W, Wu X, Shrestha P, Lei A. Long-term safety of aclidinium bromide/formoterol fumarate fixed-dose combination: Results of a randomized 1-year trial in patients with COPD. Respir Med. 2016 Jul;116:41-8. doi: 10.1016/j.rmed.2016.05.007	590	52	NCT01437540
Siler TM, Donald AC, O'Dell D, Church A, Fahy WA. A randomized, parallel-group study to evaluate the efficacy of umeclidinium/vilanterol 62.5/25 μg on health-related quality of life in patients with COPD. Int J Chron Obstruct Pulmon Dis. 2016 May 9;11:971-9. doi: 10.2147/COPD.S102962	496	12	NCT02152605
Mahler DA, Gifford AH, Satti A, Jessop N, Eckert JH, D'Andrea P, Mota F, Banerjee R. Long-term safety of glycopyrrolate: A randomized study in patients with moderate-to-severe COPD (GEM3). Respir Med. 2016 Jun;115:39-45. doi: 10.1016/j.rmed.2016.03.015	511	52	NCT01697696
Singh D, Schröder-Babo W, Cohuet G, Muraro A, Bonnet-Gonod F, Petruzzelli S, Hoffmann M, Siergiejko Z; TRIDENT study investigators. The bronchodilator effects of extrafine glycopyrronium added to combination treatment with beclometasone dipropionate plus formoterol in COPD: A randomised crossover study (the TRIDENT study). Respir Med. 2016 May;114:84-90. doi: 10.1016/j.rmed.2016.03.018	178	1	NCT01476813
Feldman G, Maltais F, Khindri S, Vahdati-Bolouri M, Church A, Fahy WA, Trivedi R. A randomized, blinded study to evaluate the efficacy and safety of umeclidinium 62.5 μg compared with tiotropium 18 μg in patients with COPD. Int J Chron Obstruct Pulmon Dis. 2016 Apr 7;11:719-30. doi: 10.2147/COPD.S102494	1,017	12	NCT02207829
Kalberg C, O'Dell D, Galkin D, Newlands A, Fahy WA. Dual Bronchodilator Therapy with Umeclidinium/Vilanterol Versus Tiotropium plus Indacaterol in Chronic Obstructive Pulmonary Disease: A Randomized Controlled Trial. Drugs R D. 2016 Jun;16(2):217-27. doi: 10.1007/s40268-016-0131-2	967	12	NCT02257385
Covelli H, Pek B, Schenkenberger I, Scott-Wilson C, Emmett A, Crim C. Efficacy and safety of fluticasone furoate/vilanterol or tiotropium in subjects with COPD at cardiovascular risk. Int J Chron Obstruct Pulmon Dis. 2015 Dec 18;11:1-12. doi: 10.2147/COPD.S91407	623	12	NCT01627327
Saito T, Takeda A, Hashimoto K, Kobayashi A, Hayamizu T, Hagan GW. Triple therapy with salmeterol/fluticasone propionate 50/250 plus tiotropium bromide improve lung function versus individual treatments in moderate-to-severe Japanese COPD patients: a randomized controlled trial - Evaluation of Airway sGaw after treatment with tripLE. Int J Chron Obstruct Pulmon Dis. 2015 Nov 4;10:2393-404. doi:	53	4	NCT01751113

10.2147/COPD.S89948			
Aalbers R, Maleki-Yazdi MR, Hamilton A, Waitere-Wijker S, Zhao Y, Amatto VC, Schmidt O, Bjermer L. Randomized, Double-Blind, Dose-Finding Study for Tiotropium when Added to Olodaterol, Administered via the Respimat® Inhaler in Patients with Chronic Obstructive Pulmonary Disease. Adv Ther. 2015 Sep;32(9):809-22. doi: 10.1007/s12325-015-0239-8	233	4	NCT01040403
Zheng J, Zhong N, Newlands A, Church A, Goh AH. Efficacy and safety of once-daily inhaled umeclidinium/vilanterol in Asian patients with COPD: results from a randomized, placebo-controlled study. Int J Chron Obstruct Pulmon Dis. 2015 Sep 2;10:1753-67. doi: 10.2147/COPD.S81053	580	24	NCT01636713
Ichinose M, Takizawa A, Izumoto T, Tadayasu Y, Hamilton AL, Kunz C, Fukuchi Y. Efficacy and safety of the long-acting β2-agonist olodaterol over 4 weeks in Japanese patients with chronic obstructive pulmonary disease. Int J Chron Obstruct Pulmon Dis. 2015 Aug 20;10:1673-83. doi: 10.2147/COPD.S86002	328	4	NCT00824382
Singh D, Worsley S, Zhu CQ, Hardaker L, Church A. Umeclidinium/vilanterol versus fluticasone propionate/salmeterol in COPD: a randomised trial. BMC Pulm Med. 2015 Aug 19;15:91. doi: 10.1186/s12890-015-0092-1	717	12	NCT01822899
Siler TM, Kerwin E, Sousa AR, Donald A, Ali R, Church A. Efficacy and safety of umeclidinium added to fluticasone furoate/vilanterol in chronic obstructive pulmonary disease: Results of two randomized studies. Respir Med. 2015 Sep;109(9):1155-63. doi: 10.1016/j.rmed.2015.06.006	619 + 620	12	NCT01957163<br>NCT02119286
Zhong N, Wang C, Zhou X, Zhang N, Humphries M, Wang L, Thach C, Patalano F, Banerji D; LANTERN Investigators. LANTERN: a randomized study of QVA149 versus salmeterol/fluticasone combination in patients with COPD. Int J Chron Obstruct Pulmon Dis. 2015 Jun 5;10:1015-26	744	26	NCT01709903
Nordenmark LH, Taylor R, Jorup C. Feasibility of Computed Tomography in a Multicenter COPD Trial: A Study of the Effect of AZD9668 on Structural Airway Changes. Adv Ther. 2015 Jun;32(6):548-66. doi: 10.1007/s12325-015-0215-3	52	12	NCT01054170
Donohue JF, Worsley S, Zhu CQ, Hardaker L, Church A. Improvements in lung function with umeclidinium/vilanterol versus fluticasone propionate/salmeterol in patients with moderate-to-severe COPD and infrequent exacerbations. Respir Med. 2015 Jul;109(7):870-81. doi: 10.1016/j.rmed.2015.04.018	706 + 697	12	NCT01817764<br>NCT01879410
Beeh KM, Westerman J, Kirsten AM, Hébert J, Grönke L, Hamilton A, Tetzlaff K, Derom E. The 24-h lung-function profile of once-daily tiotropium and olodaterol fixed-dose combination in chronic obstructive pulmonary disease. Pulm Pharmacol Ther. 2015 Jun;32:53-9. doi: 10.1016/j.pupt.2015.04.002	219	6	NCT01559116
Zheng J, de Guia T, Wang-Jairaj J, Newlands AH, Wang C, Crim C, Zhong N. Efficacy and safety of fluticasone furoate/vilanterol (50/25 mcg; 100/25 mcg; 200/25 mcg) in Asian patients with chronic obstructive pulmonary disease: a randomized placebo-controlled trial. Curr Med Res Opin. 2015 Jun;31(6):1191-200. doi: 10.1185/03007995.2015.1036016	643	24	NCT01376245
Maleki-Yazdi MR, Beck E, Hamilton AL, Korducki L, Koker P, Fogarty C. A randomised, placebo-controlled, Phase II, dose-ranging trial of once-daily treatment with olodaterol, a novel long-acting β2-	405	4	NCT00452400

agonist, for 4 weeks in patients with chronic obstructive pulmonary disease. Respir Med. 2015 May;109(5):596-605. doi: 10.1016/j.rmed.2015.02.012			
Joos GF, Aumann JL, Coeck C, Korducki L, Hamilton AL, Kunz C, Aalbers R. A randomised, double-blind, four-way, crossover trial comparing the 24-h FEV1 profile for once-daily versus twice-daily treatment with olodaterol, a novel long-acting β2-agonist, in patients with chronic obstructive pulmonary disease. Respir Med. 2015 May;109(5):606-15. doi: 10.1016/j.rmed.2015.02.005	47	12	NCT00846768
Martinez FJ, Calverley PM, Goehring UM, Brose M, Fabbri LM, Rabe KF. Effect of roflumilast on exacerbations in patients with severe chronic obstructive pulmonary disease uncontrolled by combination therapy (REACT): a multicentre randomised controlled trial. Lancet. 2015 Mar 7;385(9971):857-66. doi: 10.1016/S0140-6736(14)62410-7	1,945	52	NCT01329029
Wang C, Sun T, Huang Y, Humphries M, Bai L, Li L, Wang Q, Kho P, Firth R, D'Andrea P. Efficacy and safety of once-daily glycopyrronium in predominantly Chinese patients with moderate-to-severe chronic obstructive pulmonary disease: the GLOW7 study. Int J Chron Obstruct Pulmon Dis. 2015 Jan 5;10:57-68. doi: 10.2147/COPD.S72650. Erratum in: Int J Chron Obstruct Pulmon Dis. 2015;10:1513	460	26	NCT01566604
Buhl R, Maltais F, Abrahams R, Bjermer L, Derom E, Ferguson G, Fležar M, Hébert J, McGarvey L, Pizzichini E, Reid J, Veale A, Grönke L, Hamilton A, Korducki L, Tetzlaff K, Waitere-Wijker S, Watz H, Bateman E. Tiotropium and olodaterol fixed-dose combination versus mono-components in COPD (GOLD 2-4). Eur Respir J. 2015 Apr;45(4):969-79. doi: 10.1183/09031936.00136014	2,624 + 2,538	24	NCT01431274 NCT01431287
Feldman GJ, Bernstein JA, Hamilton A, Nivens MC, Korducki L, LaForce C. The 24-h FEV1 time profile of olodaterol once daily via Respimat® and formoterol twice daily via Aerolizer® in patients with GOLD 2-4 COPD: results from two 6-week crossover studies. Springerplus. 2014 Aug 9;3:419. doi: 10.1186/2193-1801-3-419	99 + 100	6	NCT00931385 NCT00932646
D'Urzo AD, Rennard SI, Kerwin EM, Mergel V, Leselbaum AR, Caracta CF; AUGMENT COPD study investigators. Efficacy and safety of fixed-dose combinations of aclidinium bromide/formoterol fumarate: the 24-week, randomized, placebo-controlled AUGMENT COPD study. Respir Res. 2014 Oct 14;15(1):123. doi: 10.1186/s12931-014-0123-0	1,692	24	NCT01437397
Beeh KM, Watz H, Puente-Maestu L, de Teresa L, Jarreta D, Caracta C, Garcia Gil E, Magnussen H. Aclidinium improves exercise endurance, dyspnea, lung hyperinflation, and physical activity in patients with COPD: a randomized, placebo-controlled, crossover trial. BMC Pulm Med. 2014 Dec 23;14:209. doi: 10.1186/1471-2466-14-209	112	3	NCT01471171
Maleki-Yazdi MR, Kaelin T, Richard N, Zvarich M, Church A. Efficacy and safety of umeclidinium/vilanterol 62.5/25 mcg and tiotropium 18 mcg in chronic obstructive pulmonary disease: results of a 24-week, randomized, controlled trial. Respir Med. 2014 Dec;108(12):1752-60. doi: 10.1016/j.rmed.2014.10.002	905	24	NCT01777334
Maltais F, Singh S, Donald AC, Crater G, Church A, Goh AH, Riley JH. Effects of a combination of	307 + 348	12	NCT01323660

umeclidinium/vilanterol on exercise endurance in patients with chronic obstructive pulmonary disease: two randomized, double-blind clinical trials. Ther Adv Respir Dis. 2014 Dec;8(6):169-81. doi: 10.1177/1753465814559209			NCT01328444
Singh D, Jones PW, Bateman ED, Korn S, Serra C, Molins E, Caracta C, Gil EG, Leselbaum A. Efficacy and safety of aclidinium bromide/formoterol fumarate fixed-dose combinations compared with individual components and placebo in patients with COPD (ACLIFORM-COPD): a multicentre, randomised study. BMC Pulm Med. 2014 Nov 18;14:178. doi: 10.1186/1471-2466-14-178	1,729	24	NCT01462942
Rossi A, van der Molen T, del Olmo R, Papi A, Wehbe L, Quinn M, Lu C, Young D, Cameron R, Bucchioni E, Altman P. INSTEAD: a randomised switch trial of indacaterol versus salmeterol/fluticasone in moderate COPD. Eur Respir J. 2014 Dec;44(6):1548-56. doi: 10.1183/09031936	581	26	NCT01555138
ZuWallack R, Allen L, Hernandez G, Ting N, Abrahams R. Efficacy and safety of combining olodaterol Respimat(®) and tiotropium HandiHaler(®) in patients with COPD: results of two randomized, double-blind, active-controlled studies. Int J Chron Obstruct Pulmon Dis. 2014 Oct 14;9:1133-44. doi: 10.2147/COPD.S72482	1,132 + 1,135	12	NCT01694771 NCT01696058
Watz H, Krippner F, Kirsten A, Magnussen H, Vogelmeier C. Indacaterol improves lung hyperinflation and physical activity in patients with moderate chronic obstructive pulmonary disease--a randomized, multicenter, double-blind, placebo-controlled study. BMC Pulm Med. 2014 Oct 4;14:158. doi: 10.1186/1471-2466-14-158	129	3	NCT01012765
Ohar JA, Crater GD, Emmett A, Ferro TJ, Morris AN, Raphiou I, Sriram PS, Dransfield MT. Fluticasone propionate/salmeterol 250/50 µg versus salmeterol 50 µg after chronic obstructive pulmonary disease exacerbation. Respir Res. 2014 Sep 24;15(1):105. doi: 10.1186/s12931-014-0105-2	639	26	NCT01110200
Koch A, Pizzichini E, Hamilton A, Hart L, Korducki L, De Salvo MC, Paggiaro P. Lung function efficacy and symptomatic benefit of olodaterol once daily delivered via Respimat® versus placebo and formoterol twice daily in patients with GOLD 2-4 COPD: results from two replicate 48-week studies. Int J Chron Obstruct Pulmon Dis. 2014 Jul 5;9:697-714. doi: 10.2147/COPD.S62502	904 + 934	48	NCT00793624 NCT00796653
Donohue JF, Niewoehner D, Brooks J, O'Dell D, Church A. Safety and tolerability of once-daily umeclidinium/vilanterol 125/25 mcg and umeclidinium 125 mcg in patients with chronic obstructive pulmonary disease: results from a 52-week, randomized, double-blind, placebo-controlled study. Respir Res. 2014 Jul 11;15(1):78. doi: 10.1186/1465-9921-15-78	562	52	NCT01316887
Dransfield MT, Feldman G, Korenblat P, LaForce CF, Locantore N, Pistolesi M, Watkins ML, Crim C, Martinez FJ. Efficacy and safety of once-daily fluticasone furoate/vilanterol (100/25 mcg) versus twice-daily fluticasone propionate/salmeterol (250/50 mcg) in COPD patients. Respir Med. 2014 Aug;108(8):1171-9. doi: 10.1016/j.rmed.2014.05.008	521 + 511 + 828	12	NCT01323634 NCT01323621 NCT01706328
Ferguson GT, Feldman GJ, Hofbauer P, Hamilton A, Allen L, Korducki L, Sachs P. Efficacy and safety of olodaterol once daily delivered via Respimat® in patients with GOLD 2-4 COPD: results from two replicate	624 + 642	48	NCT00782210 NCT00782509

48-week studies. Int J Chron Obstruct Pulmon Dis. 2014 Jun 16;9:629-45. doi: 10.2147/COPD.S61717			
Wedzicha JA, Singh D, Vestbo J, Paggiaro PL, Jones PW, Bonnet-Gonod F, Cohuet G, Corradi M, Vezzoli S, Petruzzelli S, Agusti A; FORWARD Investigators. Extrafine beclomethasone/formoterol in severe COPD patients with history of exacerbations. Respir Med. 2014 Aug;108(8):1153-62. doi: 10.1016/j.rmed.2014.05.013	1,199	48	NCT00929851
Troosters T, Sciurba FC, Decramer M, Siafakas NM, Klioze SS, Sutradhar SC, Weisman IM, Yunis C. Tiotropium in patients with moderate COPD naive to maintenance therapy: a randomised placebo-controlled trial. NPJ Prim Care Respir Med. 2014 May 20;24:14003. doi: 10.1038/npjpcrm.2014.3	457	24	NCT00523991
Decramer M, Anzueto A, Kerwin E, Kaelin T, Richard N, Crater G, Tabberer M, Harris S, Church A. Efficacy and safety of umeclidinium plus vilanterol versus tiotropium, vilanterol, or umeclidinium monotherapies over 24 weeks in patients with chronic obstructive pulmonary disease: results from two multicentre, blinded, randomised controlled trials. Lancet Respir Med. 2014 Jun;2(6):472-86. doi: 10.1016/S2213-2600(14)70065-7	845 + 869	24	NCT01316900<br>NCT01316913
Singh D, Nicolini G, Bindi E, Corradi M, Guastalla D, Kampschulte J, Pierzchała W, Sayiner A, Szilasi M, Terzano C, Vestbo J; FUTURE (Foster Upgrades TherapeUtic care in REspiratory disease) study group. Extrafine beclomethasone/formoterol compared to fluticasone/salmeterol combination therapy in COPD. BMC Pulm Med. 2014 Mar 12;14:43. doi: 10.1186/1471-2466-14-43	419	12	NCT01245569
Vincken W, Aumann J, Chen H, Henley M, McBryan D, Goyal P. Efficacy and safety of coadministration of once-daily indacaterol and glycopyrronium versus indacaterol alone in COPD patients: the GLOW6 study. Int J Chron Obstruct Pulmon Dis. 2014 Feb 24;9:215-28. doi: 10.2147/COPD.S51592	449	12	NCT01604278
Beeh KM, Korn S, Beier J, Jadayel D, Henley M, D'Andrea P, Banerji D. Effect of QVA149 on lung volumes and exercise tolerance in COPD patients: the BRIGHT study. Respir Med. 2014 Apr;108(4):584-92. doi: 10.1016/j.rmed.2014.01.006	85	3	NCT01294787
Chapman KR, Beeh KM, Beier J, Bateman ED, D'Urzo A, Nutbrown R, Henley M, Chen H, Overend T, D'Andrea P. A blinded evaluation of the efficacy and safety of glycopyrronium, a once-daily long-acting muscarinic antagonist, versus tiotropium, in patients with COPD: the GLOW5 study. BMC Pulm Med. 2014 Jan 17;14:4. doi: 10.1186/1471-2466-14-4	657	12	NCT01613326
Church A, Beerahee M, Brooks J, Mehta R, Shah P. Dose response of umeclidinium administered once or twice daily in patients with COPD: a randomised cross-over study. BMC Pulm Med. 2014 Jan 6;14:2. doi: 10.1186/1471-2466-14-2	163	1	NCT01372410
Mahler DA, Decramer M, D'Urzo A, Worth H, White T, Alagappan VK, Chen H, Gallagher N, Kulich K, Banerji D. Dual bronchodilation with QVA149 reduces patient-reported dyspnoea in COPD: the BLAZE study. Eur Respir J. 2014 Jun;43(6):1599-609. doi: 10.1183/09031936.00124013	247	6	NCT01490125
Decramer ML, Chapman KR, Dahl R, Frith P, Devouassoux G, Fritscher C, Cameron R, Shoaib M, Lawrence D, Young D, McBryan D; INVIGORATE investigators. Once-daily indacaterol versus tiotropium	3,444	52	NCT00845728

for patients with severe chronic obstructive pulmonary disease (INVIGORATE): a randomised, blinded, parallel-group study. Lancet Respir Med. 2013 Sep;1(7):524-33. doi: 10.1016/S2213-2600(13)70158-9			
Gelb AF, Tashkin DP, Make BJ, Zhong X, Garcia Gil E, Caracta C; LAS-MD-35 study investigators. Long-term safety and efficacy of twice-daily aclidinium bromide in patients with COPD. Respir Med. 2013 Dec;107(12):1957-65. doi: 10.1016/j.rmed.2013.07.001	605	52	NCT01044459
Dahl R, Chapman KR, Rudolf M, Mehta R, Kho P, Alagappan VK, Chen H, Banerji D. Safety and efficacy of dual bronchodilation with QVA149 in COPD patients: the ENLIGHTEN study. Respir Med. 2013 Oct;107(10):1558-67. doi: 10.1016/j.rmed.2013.05.016	339	52	NCT01120717
Donohue JF, Maleki-Yazdi MR, Kilbride S, Mehta R, Kalberg C, Church A. Efficacy and safety of once-daily umeclidinium/vilanterol 62.5/25 mcg in COPD. Respir Med. 2013 Oct;107(10):1538-46. doi: 10.1016/j.rmed.2013.06.001	1,532	24	NCT01313650
Wielders PL, Ludwig-Sengpiel A, Locantore N, Baggen S, Chan R, Riley JH. A new class of bronchodilator improves lung function in COPD: a trial with GSK961081. Eur Respir J. 2013 Oct;42(4):972-81. doi: 10.1183/09031936.00165712	436	4	NCT01319019
Chapman KR, Hurst JR, Frent SM, Larbig M, Fogel R, Guerin T, Banerji D, Patalano F, Goyal P, Pfister P, Kostikas K, Wedzicha JA. Long-Term Triple Therapy De-escalation to Indacaterol/Glycopyrronium in Patients with Chronic Obstructive Pulmonary Disease (SUNSET): A Randomized, Double-Blind, Triple-Dummy Clinical Trial. Am J Respir Crit Care Med. 2018 Aug 1;198(3):329-339. doi: 10.1164/rccm.201803-0405OC	527	26	NCT02603393
Lipson DA, Barnhart F, Brealey N, Brooks J, Criner GJ, Day NC, Dransfield MT, Halpin DMG, Han MK, Jones CE, Kilbride S, Lange P, Lomas DA, Martinez FJ, Singh D, Tabberer M, Wise RA, Pascoe SJ; IMPACT Investigators. Once-Daily Single-Inhaler Triple versus Dual Therapy in Patients with COPD. N Engl J Med. 2018 May 3;378(18):1671-1680. doi: 10.1056/NEJMoa1713901	10,355	52	NCT02164513
Zhou Y, Zhong NS, Li X, Chen S, Zheng J, Zhao D, Yao W, Zhi R, Wei L, He B, Zhang X, Yang C, Li Y, Li F, Du J, Gui J, Hu B, Bai C, Huang P, Chen G, Xu Y, Wang C, Liang B, Li Y, Hu G, Tan H, Ye X, Ma X, Chen Y, Hu X, Tian J, Zhu X, Shi Z, Du X, Li M, Liu S, Yu R, Zhao J, Ma Q, Xie C, Li X, Chen T, Lin Y, Zeng L, Ye C, Ye W, Luo X, Zeng L, Yu S, Guan WJ, Ran P. Tiotropium in Early-Stage Chronic Obstructive Pulmonary Disease. N Engl J Med. 2017 Sep 7;377(10):923-935. doi: 10.1056/NEJMoa1700228	841	104	NCT01455129
Wedzicha JA, Banerji D, Chapman KR, Vestbo J, Roche N, Ayers RT, Thach C, Fogel R, Patalano F, Vogelmeier CF; FLAME Investigators. Indacaterol-Glycopyrronium versus Salmeterol-Fluticasone for COPD. N Engl J Med. 2016 Jun 9;374(23):2222-34. doi: 10.1056/NEJMoa1516385	3,362	52	NCT01782326
Mahler DA, Kerwin E, Ayers T, FowlerTaylor A, Maitra S, Thach C, Lloyd M, Patalano F, Banerji D. FLIGHT1 and FLIGHT2: Efficacy and Safety of QVA149 (Indacaterol/Glycopyrrolate) versus Its Monocomponents and Placebo in Patients with Chronic Obstructive Pulmonary Disease. Am J Respir Crit	1,042 + 1,001	12	NCT01727141 NCT01712516

Care Med. 2015 Nov 1;192(9):1068-79. doi: 10.1164/rccm.201505-1048OC			
Lee SH, Lee J, Yoo KH, Uh ST, Park MJ, Lee SY, Kim JY, Kim DK, Kim SJ, Lee KH, Yoo CG. Efficacy and safety of aclidinium bromide in patients with COPD: A phase 3 randomized clinical trial in a Korean population. Respirology. 2015 Nov;20(8):1222-8. doi: 10.1111/resp.12641	263	12	NCT01636401
Frith PA, Thompson PJ, Ratnavadivel R, Chang CL, Bremner P, Day P, Frenzel C, Kurstjens N; Glisten Study Group. Glycopyrronium once-daily significantly improves lung function and health status when combined with salmeterol/fluticasone in patients with COPD: the GLISTEN study, a randomised controlled trial. Thorax. 2015 Jun;70(6):519-27. doi: 10.1136/thoraxjnl-2014-206670	773	12	NCT01513460
Buhl R, Gessner C, Schuermann W, Foerster K, Sieder C, Hiltl S, Korn S. Efficacy and safety of once-daily QVA149 compared with the free combination of once-daily tiotropium plus twice-daily formoterol in patients with moderate-to-severe COPD (QUANTIFY): a randomised, non-inferiority study. Thorax. 2015 Apr;70(4):311-9. doi: 10.1136/thoraxjnl-2014-206345	934	26	NCT01120717
Donohue JF, Hanania NA, Make B, Miles MC, Mahler DA, Curry L, Tosiello R, Wheeler A, Tashkin DP. One-year safety and efficacy study of arformoterol tartrate in patients with moderate to severe COPD. Chest. 2014 Dec;146(6):1531-1542. doi: 10.1378/chest.14-0117	841	52	NCT00909779
Casaburi R, Maltais F, Porszasz J, Albers F, Deng Q, Iqbal A, Paden HA, O'Donnell DE; 205.440 Investigators. Effects of tiotropium on hyperinflation and treadmill exercise tolerance in mild to moderate chronic obstructive pulmonary disease. Ann Am Thorac Soc. 2014 Nov;11(9):1351-61. doi: 10.1513/AnnalsATS.201404-174OC	126	22	NCT01072396
Magnussen H, Disse B, Rodriguez-Roisin R, Kirsten A, Watz H, Tetzlaff K, Towse L, Finnigan H, Dahl R, Decramer M, Chanez P, Wouters EF, Calverley PM; WISDOM Investigators. Withdrawal of inhaled glucocorticoids and exacerbations of COPD. N Engl J Med. 2014 Oct 2;371(14):1285-94. doi: 10.1056/NEJMoa1407154	2,485	52	NCT00975195
Celli B, Crater G, Kilbride S, Mehta R, Tabberer M, Kalberg CJ, Church A. Once-daily umeclidinium/vilanterol 125/25 mcg in COPD: a randomized, controlled study. Chest. 2014 May;145(5):981-991. doi: 10.1378/chest.13-1579	1,493	24	NCT01313637
Hohlfeld JM, Sharma A, van Noord JA, Cornelissen PJ, Derom E, Towse L, Peterkin V, Disse B. Pharmacokinetics and pharmacodynamics of tiotropium solution and tiotropium powder in chronic obstructive pulmonary disease. J Clin Pharmacol. 2014 Apr;54(4):405-14. doi: 10.1002/jcph.215	154	4	NCT01222533
Yao W, Wang C, Zhong N, Han X, Wu C, Yan X, Chen P, Yang W, Henley M, Kramer B. Effect of once-daily indacaterol in a predominantly Chinese population with chronic obstructive pulmonary disease: a 26-week Asia-Pacific study. Respirology. 2014 Feb;19(2):231-238. doi: 10.1111/resp.12211	561	26	NCT00792805
Zheng J, Yang J, Zhou X, Zhao L, Hui F, Wang H, Bai C, Chen P, Li H, Kang J, Brose M, Richard F, Goehring UM, Zhong N. Roflumilast for the treatment of COPD in an Asian population: a randomized, double-blind, parallel-group study. Chest. 2014 Jan;145(1):44-52. doi: 10.1378/chest.13-1252	626	24	NCT01313494

Rennard SI, Scanlon PD, Ferguson GT, Rekeda L, Maurer BT, Garcia Gil E, Caracta CF. ACCORD COPD II: a randomized clinical trial to evaluate the 12-week efficacy and safety of twice-daily aclidinium bromide in chronic obstructive pulmonary disease patients. Clin Drug Investig. 2013 Dec;33(12):893-904. doi: 10.1007/s40261-013-0138-1	544	12	NCT01045161
Wise RA, Anzueto A, Cotton D, Dahl R, Devins T, Disse B, Dusser D, Joseph E, Kattenbeck S, Koenen-Bergmann M, Pledger G, Calverley P; TIOSPIR Investigators. Tiotropium Respimat inhaler and the risk of death in COPD. N Engl J Med. 2013 Oct 17;369(16):1491-501. doi: 10.1056/NEJMoa1303342	17,135	137	NCT01126437
Cooper CB, Celli BR, Jardim JR, Wise RA, Legg D, Guo J, Kesten S. Treadmill endurance during 2-year treatment with tiotropium in patients with COPD: a randomized trial. Chest. 2013 Aug;144(2):490-497. doi: 10.1378/chest.12-2613	519	96	NCT00525512
Singh D, Ferguson GT, Bolitschek J, Grönke L, Hallmann C, Bennett N, Abrahams R, Schmidt O, Bjermer L. Tiotropium + olodaterol shows clinically meaningful improvements in quality of life. Respir Med. 2015 Oct;109(10):1312-9. doi: 10.1016/j.rmed.2015.08.002	814 + 809	12	NCT01964352<br>NCT02006732
Mannino DM, Clerisme-Beaty EM, Franceschina J, Ting N, Leidy NK. Exacerbation recovery patterns in newly diagnosed or maintenance treatment-naïve patients with COPD: secondary analyses of TICARI 1 trial data. Int J Chron Obstruct Pulmon Dis. 2018 May 10;13:1515-1525. doi: 10.2147/COPD.S149669	140	12	NCT01483625
Zhong N, Zheng J, Lee SH, Lipson DA, Du X, Wu S. Efficacy and Safety of Once-Daily Inhaled Umeclidinium in Asian Patients with COPD: Results from a Randomized, Placebo-Controlled Study. Int J Chron Obstruct Pulmon Dis. 2020 Apr 17;15:809-819. doi: 10.2147/COPD.S215011	306	24	NCT02184611
Ferguson GT, Feldman G, Pudi KK, Barnes CN, Moran EJ, Haumann B, Pendyala S, Crater G. Improvements in Lung Function with Nebulized Revefenacin in the Treatment of Patients with Moderate to Very Severe COPD: Results from Two Replicate Phase III Clinical Trials. Chronic Obstr Pulm Dis. 2019 Apr 9;6(2):154-165. doi: 10.15326/jcopdf.6.2.2018.0152	619 + 611	12	NCT02512510<br>NCT02459080
Beeh KM, Emirova A, Prunier H, Santoro D, Nandeuil MA. Dose-response of an extrafine dry powder inhaler formulation of glycopyrronium bromide: randomized, double-blind, placebo-controlled, dose-ranging study (GlycoNEXT). Int J Chron Obstruct Pulmon Dis. 2018 May 25;13:1701-1711. doi: 10.2147/COPD.S168493	202	4	NCT02680197
Watz H, Bagul N, Rabe KF, Rennard S, Alagappan VK, Román J, Facius A, Calverley PM. Use of a 4-week up-titration regimen of roflumilast in patients with severe COPD. Int J Chron Obstruct Pulmon Dis. 2018 Mar 6;13:813-822. doi: 10.2147/COPD.S154012	1,321	12	NCT02165826
Donohue JF, Goodin T, Tosiello R, Wheeler A. Dose selection for glycopyrrolate/eFlow® phase III clinical studies: results from GOLDEN (Glycopyrrolate for Obstructive Lung Disease via Electronic Nebulizer) phase II dose-finding studies. Respir Res. 2017 Dec 4;18(1):202. doi: 10.1186/s12931-017-0681-z	282 + 96	4/1	NCT01706536<br>NCT02038829
Reisner C, Gottschlich G, Fakih F, Koser A, Krainson J, Delacruz L, Arora S, Feldman G, Pudi K, Siddiqui S, Orevillo C, Maes A, St Rose E, Martin U. 24-h bronchodilation and inspiratory capacity improvements	80 + 43	4	NCT02347072<br>NCT02347085

with glycopyrrolate/formoterol fumarate via co-suspension delivery technology in COPD. Respir Res. 2017 Aug 18;18(1):157. doi: 10.1186/s12931-017-0636-4			
Singh D, Scuri M, Collarini S, Vezzoli S, Mariotti F, Muraro A, Acerbi D. Bronchodilator efficacy of extrafine glycopyrronium bromide: the Glyco 2 study. Int J Chron Obstruct Pulmon Dis. 2017 Jul 7;12:2001-2014. doi: 10.2147/COPD.S137659	38	1	NCT01176903
Tashkin DP, Martinez FJ, Rodriguez-Roisin R, Fogarty C, Gotfried M, Denenberg M, Gottschlich G, Donohue JF, Orevillo C, Darken P, St Rose E, Strom S, Fischer T, Golden M, Reisner C. A multicenter, randomized, double-blind dose-ranging study of glycopyrrolate/formoterol fumarate fixed-dose combination metered dose inhaler compared to the monocomponents and open-label tiotropium dry powder inhaler in patients with moderate-to-severe COPD. Respir Med. 2016 Nov;120:16-24. doi: 10.1016/j.rmed.2016.09.012	132	1	NCT01587079
Agustí A, de Teresa L, De Backer W, Zvarich MT, Locantore N, Barnes N, Bourbeau J, Crim C. A comparison of the efficacy and safety of once-daily fluticasone furoate/vilanterol with twice-daily fluticasone propionate/salmeterol in moderate to very severe COPD. Eur Respir J. 2014 Mar;43(3):763-72. doi: 10.1183/09031936.00054213	528	12	NCT01342913
Trivedi R, Richard N, Mehta R, Church A. Umeclidinium in patients with COPD: a randomised, placebo-controlled study. Eur Respir J. 2014 Jan;43(1):72-81. doi: 10.1183/09031936.00033213	206	12	NCT01387230
D'Urzo A, Kerwin E, Rennard S, He T, Gil EG, Caracta C. One-year extension study of ACCORD COPD I: safety and efficacy of two doses of twice-daily aclidinium bromide in patients with COPD. COPD. 2013 Aug;10(4):500-10. doi: 10.3109/15412555.2013.791809	291	52	NCT00970268
Donohue JF, Singh D, Munzu C, Kilbride S, Church A. Magnitude of umeclidinium/vilanterol lung function effect depends on monotherapy responses: Results from two randomised controlled trials. Respir Med. 2016 Mar;112:65-74. doi: 10.1016/j.rmed.2016.01.001	207 + 182	2	NCT02014480<br>NCT01716520
Beeh KM, Derom E, Echave-Sustaeta J, Grönke L, Hamilton A, Zhai D, Bjermer L. The lung function profile of once-daily tiotropium and olodaterol via Respimat(®) is superior to that of twice-daily salmeterol and fluticasone propionate via Accuhaler(®) (ENERGITO(®) study). Int J Chron Obstruct Pulmon Dis. 2016 Feb 4;11:193-205. doi: 10.2147/COPD.S95055	220	6	NCT01969721
Dahl R, Jadayel D, Alagappan VK, Chen H, Banerji D. Efficacy and safety of QVA149 compared to the concurrent administration of its monocomponents indacaterol and glycopyrronium: the BEACON study. Int J Chron Obstruct Pulmon Dis. 2013;8:501-8. doi: 10.2147/COPD.S49615	193	4	NCT01529632
Pudi KK, Barnes CN, Moran EJ, Haumann B, Kerwin E. A 28-day, randomized, double-blind, placebo-controlled, parallel group study of nebulized revefenacin in patients with chronic obstructive pulmonary disease. Respir Res. 2017 Nov 2;18(1):182. doi: 10.1186/s12931-017-0647-1	355	4	NCT02040792

Table S2. Final model parameter estimates with their uncertainty

Parameter	Estimate (SE) [shrinkage SD%][a]	
	**Data 1996 – 2013** [7]	**Augmented data (1996 – 2021)**
Typical baseline FEV1 (L)	1.17 (0.017)	1.17 (0.012)
Disease progression slope (L/year)	0.0359 (0.004)	0.032 (0.003)
Placebo E_{max}	0.0002 (0.005)	-0.009 (0.005)
Log of time for half maximum placebo effect (weeks)	2.41 (0.21)	3.0 (0.40)
Log of onset rate of anti-inflammatory treatments (weeks)	-0.563 (0.42)	-0.63 (0.25)
Reference efficacy of budesonide 160 ug b.i.d. (L)	0.044 (0.007)	0.031 (0.013)
Efficacy of fluticasone b.i.d. (L)	0.0524 (0.006)	0.0423[b] (0.005)
Reference efficacy of formoterol 9 ug b.i.d. (L)	0.0706 (0.009)	0.0706 (0.006)
Reference efficacy of indacaterol 75 ug q.d. (L)	0.129 (0.006)	0.128 (0.008)
Efficacy of mometasone q.d. (L)	0.0659 (0.007)	0.0688 (0.007)
Reference efficacy of roflumilast 500 ug q.d. (L)	0.0791 (0.007)	0.0786 (0.012)
Efficacy of salmeterol b.i.d. (L)	0.0842 (0.007)	0.0793 (0.005)
Reference efficacy of tiotropium (blinded) 18 ug q.d. (Handihaler) (L)	0.129 (0.004)	0.122 (0.005)
Reference efficacy of tiotropium (blinded) 5 ug q.d. (Respimat) (L)	0.134 (0.007)	0.120 (0.004)
Fractional bronchodilator effect for postSA bronchodilator measurements	0.742 (0.267)	0.525 (0.14)
Reference efficacy of aclidinium 200 ug q.d. (L)	0.0709 (0.007)	0.0712 (0.008)
Efficacy of beclomethasone (L)	0.0303 (0.004)	0.0577 (0.013)
Efficacy of cilomilast (L)	0.0457 (0.003)	0.0427 (0.005)
Reference efficacy of glycopyrronium 100 ug q.d. (L)	0.135 (0.006)	0.131 (0.006)
LABD interaction parameter	1.42 (0.092)	1.35 (0.044)
Covariate effect of disease severity on baseline	-0.167 (0.0146)	-0.163 (0.01)
Covariate effect of exacerbation history on baseline	-0.0402 (0.0172)	-0.0397 (0.01)
Log of ED_{50} for tiotropium (Handihaler)	1.44 (0.429)	0.845 (0.52)
Log of ED_{50} for indacaterol	3.19 (0.338)	1.68 (2.03)

Log of ED_{50} for aclidinium q.d.	4.12 (0.178)	4.02 (0.20)
Onset rate for q.d. LABA bronchodilator (weeks)	9.87 (2.02)	9.19 (1.65)
Log of ISV CV for the disease progression slope	-0.969 (1.23)	-0.611 (0.20)
Log of ED_{50} for roflumilast	5.75 (0.286)	5.09 (1.16)
ISV CV for anti-inflammatory efficacy	0.227 (0.098)	0.405 (0.10)
Log of fraction of studies with immediate placebo response	-0.729 (0.212)	-0.804 (0.16)
Interaction parameter for COPD medication history with lower disease severity	0.307 (0.0965)	0.196 (0.06)
Effect of predicted baseline <1.2 L on anti-inflammatory efficacy	1.39 (0.391)	0.638 (0.24)
Covariate effect of age on baseline	-0.012 (0.003)	-0.0091 (0.002)
Effect of predicted baseline <1.2 L on bronchodilator efficacy	0.237 (0.0794)	0.268 (0.09)
Reference efficacy of aclidinium 400 ug b.i.d. (L)	0.120 (0.01)	0.0981 (0.007)
Log of ED_{50} for aclidinium b.i.d.	5.54 (0.479)	5.25 (0.49)
Relative efficacy of mometasone b.i.d. compared to q.d.	0.832 (0.095)	0.818 (0.09)
Relative reference efficacy of fluticasone 200 ug q.d. compared to b.i.d	0.753 (0.259)	0.0436[c] (0.005)
Efficacy of umeclidinium q.d. (L)	0.127 (0.006)	0.144 (0.006)
Efficacy of GSK23305 (L)	0.211 (0.011)	0.193 (0.006)
Reference efficacy of vilanterol 25 ug q.d. (L)	0.139 (0.008)	0.114 (0.004)
Efficacy of BEA2180 (L)	0.118 (0.007)	0.104 (0.003)
Efficacy of PH797804 (L)	0.0753 (0.0129)	0.0925 (0.01)
Efficacy of AZD9668 (L)	0.0213 (0.003)	0.0122 (0.01)
Relative efficacy of tiotropium (open-label) 18 ug q.d. (Spiriva) compared to blinded administration	0.930 (0.065)	0.952 (0.05)
Log of ED_{50} for glycopyrronium	2.72 (0.355)	2.31 (0.40)
Log of ED_{50} for vilanterol	0.725 (0.163)	0.709 (0.10)
ISV CV for bronchodilator efficacy	0.125 (0.043)	0.185 (0.03)
Onset rate for q.d. LAAC bronchodilators (weeks)	16.4 (3.94)	12.1 (2.28)

Log of ED_{50} for fluticasone q.d.	1.93 (3.46)	2.43[d] (1.99)
Log of ED_{50} for budesonide	3.89 (2.16)	5.83 (1.96)
Log of ED_{50} for formoterol	2.38 (0.629)	1.10 (1.41)
Post-bronchodilator correction	-	0.889 (0.007)
Log of ED_{50} for revefenacin	-	3.86 (0.367)
Reference efficacy of revefenacin 175 ug q.d. (L)	-	0.144 (0.008)
Log of ED_{50} for olodaterol	-	0.386 (0.417)
Reference efficacy of olodaterol 5 ug q.d. (L)	-	0.0893 (0.003)
Log of ED_{50} for batefenterol	-	3.0 (0.985)
Reference efficacy of batefenterol 400 ug q.d. (L)	-	0.190 (0.013)
Efficacy of olodaterol b.i.d. (L)	-	0.111 (0.002)
Efficacy of batefenterol b.i.d.	-	0.207 (0.006)
ISV variance for the typical baseline	0.0101 (0.0016)	0.0099 (0.001) [1.37]
ISV for the disease progression slope estimated as a fixed effect	1 fixed	1 fixed [62.7]
ISV variance for the placebo E_{max}	0.00123 (0.0002)	0.0021 (0.0005) [38.5]
ISV variance for the bronchodilator efficacy estimated as fixed effect	1 fixed	1 fixed [31.5]
ISV variance for the anti-inflammatory efficacy estimated as fixed effect	1 fixed	1 fixed [65.3]
Variance for imputation of age	4 fixed	4 fixed [98.8]
ISV variance for the residual error	0 fixed	0 fixed
IAV variance for the typical baseline in a study-arm with 200 patients	0.00055 (0.00009)	0.0004 (0.00006) [31.2]
Variance for imputation of ICS medication history	0.7 fixed	0.7 fixed [98.4]
Variance for imputation of LABA medication history	0.7 fixed	0.7 fixed [98.8]
Variance for imputation of LAAC medication history	1 fixed	1 fixed [99.6]
Variance for imputation of ICS background treatment	0.5 fixed	0.5 fixed [95.6]
Variance for imputation of LABA background treatment	1 fixed	1 fixed [90.0]
Variance for imputation of LAAC background treatment	0.1 fixed	0.1 fixed [97.5]

Variance of the additive residual error	0.0445 (0.005)	0.042 (0.003) [20.0]

a: reported shrinkage corresponds to the largest subpopulation of 218 studies (normal E_{max} placebo effect) from the mixture model; b: for this analysis this correspond to fluticasone propionate; c: for this analysis, this corresponds to reference efficacy of fluticasone furoate 100 ug q.d.; d: for this analysis this corresponds to fluticasone furoate
